# A Set of Experimentally Validated Decoys for the Human CC Chemokine Receptor 7 (CCR7) Obtained by Virtual Screening

**DOI:** 10.3389/fphar.2022.855653

**Published:** 2022-03-18

**Authors:** Matic Proj, Steven De Jonghe, Tom Van Loy, Marko Jukič, Anže Meden, Luka Ciber, Črtomir Podlipnik, Uroš Grošelj, Janez Konc, Dominique Schols, Stanislav Gobec

**Affiliations:** ^1^ Department of Pharmaceutical Chemistry, Faculty of Pharmacy, University of Ljubljana, Ljubljana, Slovenia; ^2^ Laboratory of Virology and Chemotherapy, Department of Microbiology, Immunology and Transplantation, Rega Institute for Medical Research, KU Leuven, Leuven, Belgium; ^3^ Faculty of Chemistry and Chemical Engineering, Laboratory of Physical Chemistry and Chemical Thermodynamics, University of Maribor, Maribor, Slovenia; ^4^ Faculty of Mathematics, Natural Sciences and Information Technologies, University of Primorska, Koper, Slovenia; ^5^ Faculty of Chemistry and Chemical Technology, University of Ljubljana, Ljubljana, Slovenia; ^6^ National Institute of Chemistry, Ljubljana, Slovenia

**Keywords:** virtual screening, decoys, chemical library, computer-aided drug design, CCR7, GPCR

## Abstract

We present a state-of-the-art virtual screening workflow aiming at the identification of novel CC chemokine receptor 7 (CCR7) antagonists. Although CCR7 is associated with a variety of human diseases, such as immunological disorders, inflammatory diseases, and cancer, this target is underexplored in drug discovery and there are no potent and selective CCR7 small molecule antagonists available today. Therefore, computer-aided ligand-based, structure-based, and joint virtual screening campaigns were performed. Hits from these virtual screenings were tested in a CCL19-induced calcium signaling assay. After careful evaluation, none of the *in silico* hits were confirmed to have an antagonistic effect on CCR7. Hence, we report here a valuable set of 287 inactive compounds that can be used as experimentally validated decoys.

## Graphical Abstract

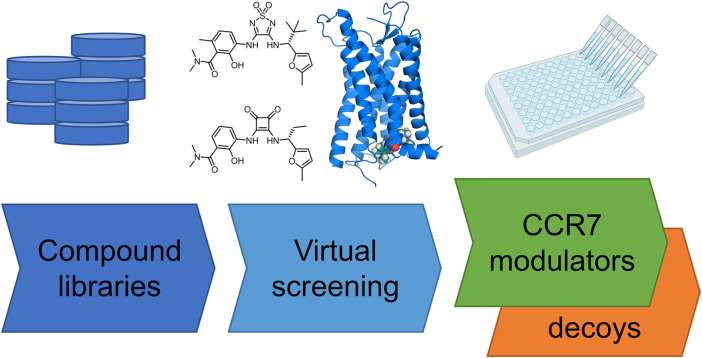

## 1 Introduction

Chemokines (chemoattractant cytokines) are small, secreted proteins (∼10 kDa) that were first described as essential mediators of immune cell migration throughout the human body. They are characterized by conserved N-terminal cysteine (C) residues (i.e., C, CC, CXC, and CX3C chemokines, where X is a variable amino acid) and exert their function through activation of G protein-coupled receptors (GPCRs). About 20 human chemokine receptors and approximately 50 different human chemokines are known. A given chemokine receptor can sometimes be activated by more than one chemokine and, at the same time, a particular chemokine can signal via multiple receptors (Griffith et al., 2014).

The CC chemokine receptor 7 (CCR7) is crucial for lymphoid organogenesis and the recruitment of naïve T lymphocytes and activated dendritic cells towards the lymph nodes, where they initiate the immune response (Zlotnik et al., 2011). CCR7 can be activated by two receptor ligands, the chemokines CCL19 and CCL21 that bind with high affinity to CCR7 (Sullivan et al., 1999). Unlike CCL19, CCL21 harbors an extended and highly positively charged C-terminal tail that mediates strong binding to glycosaminoglycans (GAGs) expressed at the cell surface (Barmore et al., 2016). Several studies revealed the biased signaling properties of CCL19 and CCL21 and indicate that both chemokines differentially target CCR7 in terms of G protein activation, β-arrestin recruitment and receptor internalization (Kohout et al., 2004; Corbisier et al., 2015; Hjortø et al., 2016). CCR7 signaling can contribute to the progression of severe human diseases. Tumor cells of diverse origins can hijack CCR7-mediated migration to metastasize, primarily to the lymph nodes (Zlotnik et al., 2011; Jørgensen et al., 2018). Recruitment of leukaemic T cells to the central nervous system is also dependent on CCR7 (Buonamici et al., 2009). Other human diseases associated with CCR7 signaling include chronic inflammatory diseases (e.g., rheumatoid arthritis) (Moschovakis and Förster, 2012). Hence, CCR7 has emerged as a promising therapeutic target, but remains understudied from a drug discovery perspective.

**FIGURE 1:**
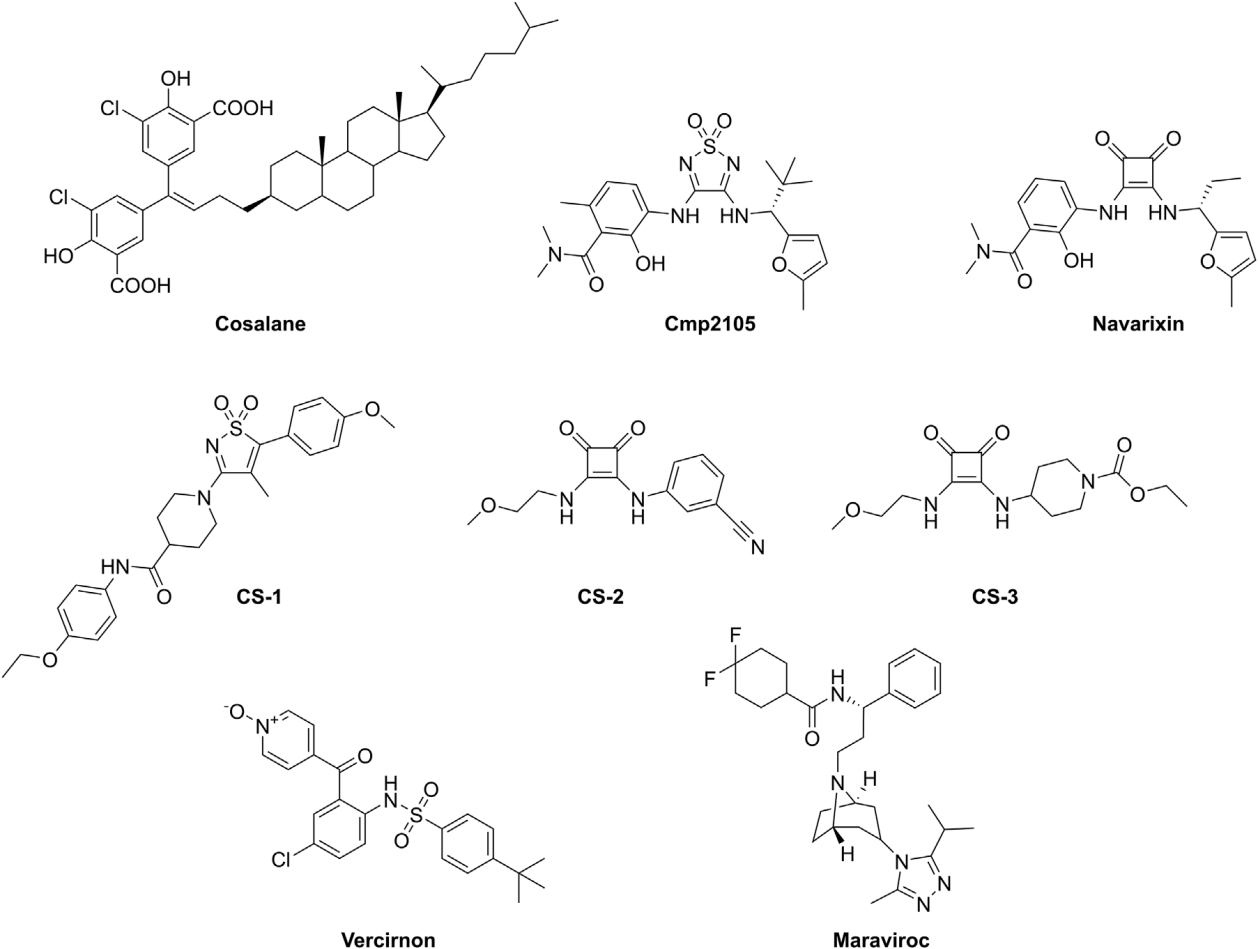
Compounds studied as CCR7 antagonists.

Even though CCR7 is implicated in various human diseases, to the best of our knowledge, no selective and potent small molecule antagonists for CCR7 have been developed so far. Recently, a high-throughput screening of 150,000 compounds using Chinese hamster ovary (CHO)–K1 cells expressing human or murine CCR7 in a β-arrestin recruitment assay was described (Hull-Ryde et al., 2018). The most potent CCR7 antagonist that emerged from this campaign was **cosalane** (Figure 1) with a half maximal inhibitory concentration (IC_50_) value of 0.2 µM (when CCL19 was used as the natural CCR7 ligand) and 2.7 µM (when CCL21 was used as the agonist). In addition, **cosalane** exhibited nearly identical activity against the human and murine CCR7 orthologues. However, the high lipophilicity of **cosalane** and its complex chemical structure make it unattractive as lead structure for further chemical optimization. Recently, the X-ray co-crystal structure of CCR7, complexed with **cmp2105** (Figure 1), was solved (Jaeger et al., 2019). This compound was shown to bind to a conserved allosteric Gi protein binding pocket at the intracellular side of the receptor. Validation of its CCR7 binding was performed in a membrane-based competition binding experiment with radiolabeled CCL19, in which an IC_50_ value of 35 nM was determined for **cmp2105**. Furthermore, CCR7 antagonism of **cmp2105** was confirmed in a cell-based β-arrestin recruitment assay, which yielded an IC_50_ value of 7.3 µM (Jaeger et al., 2019). **Cmp2105** was initially discovered by screening in a CCR7 thermal-shift assay. **Navarixin** (Figure 1) also displayed a thermostabilizing effect in this assay and subsequently an IC_50_ value of 33.9 µM was determined in the β-arrestin recruitment assay (Jaeger et al., 2019). Other analogues (i.e., **CS-1**, **CS-2**, and **CS-3**, Figure 1) also proved to be hits in the thermofluor stability assay, albeit less potent than **cmp2105** and **navarixin**, and were not further pharmacologically validated (Jaeger et al., 2019). Other known chemokine receptor ligands, such as **vercirnon** (a CCR9 antagonist) and **maraviroc** (a CCR5 antagonist) completely lacked the ability to thermally stabilize CCR7 (Jaeger et al., 2019).

To improve our understanding of the role of CCR7 in various pathologies, there is a clear need for potent, drug-like, and selective CCR7 antagonists that can be used as chemical probes to validate CCR7 as a drug target. In addition, these chemical tools can be used as starting points for medicinal chemistry-based optimization campaigns. In this study, we describe a virtual screening workflow, followed by experimental validation, in search for novel CCR7 small molecule antagonists. Known CCR7 ligands from the patent and scientific literature, whose CCR7 antagonism was independently confirmed, were used as starting points for ligand-based virtual screening (LBVS) protocols. In addition, the recently published crystal structure of CCR7 was used to perform molecular docking and to generate a pharmacophore model.

## 2 Materials and Methods

### 2.1 GPCR Assays

#### 2.1.1 CCR7 Competition Binding Assay

Human U87 glioblastoma cells that stably express CD4 and the human CCR7 receptor (U87.CD4.CCR7) were used to determine CCR7 binding affinity, essentially by adopting a previously published protocol used to study binding to another chemokine GPCR, CXCR4 (Schoofs et al., 2018). U87.CD4 cells that do not overexpress CCR7 were used as control cells to evaluate the level of non-specific cell binding of the fluorescently labeled ligand (Alexa-Fluor647 labeled CCL19, CCL19^AF647^, Almac, United Kingdom). In brief, U87.CD4.CCR7 cells were pre-incubated with compound (at different concentrations) in 150 μL assay buffer [Hank’s balanced salt solution (HBSS), 20 mM HEPES and 0.5% Fetal Calf Serum] for 15 min at room temperature (RT) in the dark. Afterwards, 50 μL of CCL19^AF647^ was added (25 ng/mL final concentration) and samples were incubated for another 30 min at RT, protected from light. Then, cells were washed twice with assay buffer and fixed in 1% paraformaldehyde in Dulbecco’s phosphate-buffered saline (DPBS).

Samples were immediately analyzed by flow cytometry (FACS Canto II, BD). Data were analysed using Flowjo. The percentage inhibition of CCL19^AF647^ binding was calculated according to formula {1—[(MFI_X_–MFI_NC_)/(MFI_PC_–MFI_NC_)]} x 100, where MFI_X_ is the mean fluorescence intensity (MFI) of the compound-treated sample, MFI_NC_ the MFI of the negative control (i.e., autofluorescence of untreated and unlabeled cells) and MFI_PC_ the MFI of the positive control (i.e., cells exposed to CCL19^AF647^ only). IC_50_ values (i.e., the compound concentration that inhibits CCL19^AF647^ binding by 50%) were calculated using four parameter non-linear curve fitting in GraphPad Prism 9.0.2. For each experiment the stain index (SI) was calculated as the ratio of the separation between MFI_PC_ and MFI_NC_, divided by two times the standard deviation of MFI_NC_.

#### 2.1.2 Calcium Mobilization Assays

U87.CD4 cells that stably express either human CCR7, CXCR2, CCR5 or CXCR4 were seeded (20,000 cells/well) in gelatin-coated black-walled polystyrene 96-well plates with clear bottom and incubated overnight at 37°C and 5% CO_2_. The next day, a fluorescent Ca^2+^-sensitive dye solution (Fluo-2 AM) was prepared as described before (Claes et al., 2018). Culture medium was removed, and cells were incubated for 45 min at room temperature in the dark. Meanwhile, 96-well polypropylene plates containing 5-fold concentrated compound dilutions and 5-fold concentrated solution of chemokine ligands (CCL19, CXCL8, LD78-β, CXCL12, respectively; all purchased from PeproTech) were prepared for use with the FLIPR Tetra device (Molecular Devices) as described before (Claes et al., 2018). The antagonistic properties of the compounds were calculated based on their capacity to inhibit the Ca^2+^ release induced by a fixed concentration of chemokine (i.e. 50 ng/mL final concentration for CCL19, CXCL8 and CXCL12 and 100 ng/ml for LD78-β), as described (Claes et al., 2018). Exactly the same protocol was used to record calcium responses in Chinese hamster ovary (CHO)-K1 cells, upon stimulation with adenosine triphosphate (ATP, purchased from Sigma).

### 2.2 Preparation of Chemical Libraries

#### 2.2.1 Active Compounds and Generated Decoys

The survey of patent and scientific literature revealed the existence of eight CCR7 antagonists (Supplementary Figure S1). Based on this, a set of 600 decoy molecules (see Supporting Excel file) was generated using DUD-E server (Mysinger et al., 2012). The generated decoys have similar physicochemical properties (molecular weight, estimated water–octanol partition coefficient (miLogP), rotatable bonds, hydrogen bond acceptors, hydrogen bond donors, and net charge), but have a different 2D topology when compared to the active compounds. Decoys can be used as alternatives to experimentally confirmed inactive compounds for the purpose of model validation.

Primary literature search identified an additional 104 compounds active on multiple chemokine receptors, namely CCR1, CCR2, CCR3, CCR4, CCR5, CCR7, CCR8, CCR9, CCR10, CXCR1, CXCR2, CXCR3, CXCR4, and CXCR7 (Supplementary Table S1). Those compounds were used to construct a focused chemokine receptor targeted compound library, as described below.

#### 2.2.2 FKKTlib Academic Compound Library

The FKKTlib academic compound library currently contains 3,428 unique synthesized compounds resulting from many years of research across various projects at the University of Ljubljana, Faculty of Chemistry and Chemical Technology. Most of the compounds in this library are heterocycles that are documented in the scientific literature. Most of the samples are available as solids and are stored in cryogenic vials labelled with a QR code that allows for quick retrieval of the samples. To ensure the stability of the samples, they are stored under argon at –25°C. Information about the compounds in the library is stored in a web-based, fully retrievable molecular structure database based on the open-source solution MolDB6, developed by Prof. Norbert Haider from the University of Vienna (Haider, 2010). The system uses MySQL as a database engine, and the molecular structures with their corresponding data are stored in MySQL tables. The check/matchmol programme is used for structure or substructure searches, which is performed in a two-step procedure: pre-selection by fingerprint matching, followed by a complete atom-by-atom comparison of the remaining candidates. Structures and data can be added via the web interface or by importing from an MDL SD file using a Perl script on the server. The library is freely accessible at: https://knjiznica-spojin.fkkt.uni-lj.si/fkktlib/.

#### 2.2.3 ZINC Library

The ZINC in-stock subset (Sterling and Irwin, 2015), containing 13.7 million drug-like compounds, was used for the virtual screening using the Ligand Similarity Using Clique Algorithm (LiSiCA) software (Lešnik et al., 2015). The ZINC subset was first filtered using the FILTER 3.1.2.2 software (OpenEye Scientific Software, Inc, Santa Fe, NM, United States; www.eyesopen.com), eliminating known or predicted aggregators, compounds containing metals, and compounds with reactive functional groups, and retaining only compounds with appropriate molecular weights (200–800 Da) and partition coefficients (-4.0-6.9) (see Supplementary Material for FILTER configuration file). The filtered ZINC library contained 8.9 million compounds. Finally, the stereoisomer and conformational model generator OMEGA 3.1.2.2 (OpenEye Scientific Software, Inc, Santa Fe, NM, United States; www.eyesopen.com) was used to enumerate stereocenters and to generate up to 30 conformers per compound.

#### 2.2.4 Chemokine Receptor Targeted Compound Library

The ZINC in-stock subset (Sterling and Irwin, 2015) was also used for the construction of a library covering compounds targeting chemokine receptors. FP2 molecular fingerprints were calculated for the 104 compounds targeting various chemokine receptors (details in Supplementary Material) as well as for the complete ZINC subset. Using OpenBabel (v2.3.0), a similarity search in ZINC was carried out with 104 queries and a Tanimoto index of ≥0.5 to obtain a similarity library of 951,471 unique structures. The similarity library was then filtered using the FILTER software (OpenEye Scientific Software, Inc, Santa Fe, NM, United States; www.eyesopen.com), as described beforehand, to obtain a focused chemokine receptor library of 539,814 compounds. Finally, OMEGA (OpenEye Scientific Software, Inc, Santa Fe, NM, United States; www.eyesopen.com) was used to enumerate all possible stereocenters and generate up to 10,000 conformers per compound (RMS of 0.3).

#### 2.2.5 MolPort Library

The second library was prepared from the MolPort database of in-stock compounds (7.5 million). It was used for core motif substructure searches, virtual screening with ROCS, docking with FRED, Glide, and ProBiS-Dock. Duplicates were removed using OpenBabel 2.4.1 (O’Boyle et al., 2011), and the database was processed using the FILTER 3.1.2.2 software (OpenEye Scientific Software, Inc, Santa Fe, NM, United States; www.eyesopen.com), eliminating known or predicted aggregators, compounds containing metals, and compounds with reactive functional groups, retaining only compounds with appropriate molecular weights (200–800 Da) and partition coefficients (-4.0–6.9) (see Supplementary Material for FILTER configuration file). Compounds known to cause interference in assay systems (Dahlin et al., 2015) were removed using the RDKit Molecule Catalog Filter node (catalog PAINS A) (RDKit: Open-source cheminformatics, 2021) as implemented in the KNIME platform (Berthold et al., 2007). Compounds with reactive functional groups (Brenk et al., 2008) were also removed. Protonation states at pH 7.4 were generated using OpenBabel 2.4.1 (O’Boyle et al., 2011). The final library contained 3.5 million compounds. Finally, the stereoisomer and conformational model generator OMEGA 3.1.2.2 (OpenEye Scientific Software, Inc, Santa Fe, NM, United States; www.eyesopen.com) was used to enumerate stereocenters and generate up to 200 conformers per compound.

#### 2.2.6 Diversity Set of Compounds Available From Trusted Commercial Vendors

The third library used for pharmacophore-based screening contained 1.1 million compounds based on curated diversity sets from Asinex, ChemBridge, ChemDiv, Enamine, KeyOrganics, and Pharmeks. The libraries were downloaded in SDF format, merged, and duplicates removed using the LigandScout database Merger and Duplicates Remover nodes implemented in the Inte:Ligand Expert KNIME Extensions. Protonation states at pH 7.4 were generated using OpenBabel 2.4.1 (O’Boyle et al., 2011). Finally, a maximum of 200 conformations were generated for each molecule using the iCon algorithm of LigandScout (Poli et al., 2018) with default “BEST” settings and saved in LDB (LigandScout database format) using the idbgen algorithm.

### 2.3 Core Motif Substructure Search

Core motif substructure searches were performed using SMILES filters applied to the MolPort library and the FKKTlib. The core motifs of cyclobutenedione (with an additional nitrogen atom) and thiourea were defined by SMILES expressions O=C1C=C(N)C1=O and NC(N)=S, respectively. First, MolPort library filtering was performed using the RDKit substructure filter node (RDKit: Open-source cheminformatics, 2021) as implemented in the KNIME analytics platform (Berthold et al., 2007). 2,452 cyclobutenediones were extracted from the MolPort database and docked to the prepared CCR7 receptor using Glide XP (Schrödinger Suite 2020-2, Schrödinger, LLC, New York, NY, 2020) (Friesner et al., 2006) as described below. Of the 100 highest scoring compounds, 16 diverse compounds were selected for purchase. Second, the MolPort database was searched for the thiourea core motif, which yielded more than 90k available compounds. Duplicates, PAINS (Dahlin et al., 2015), and compounds with reactive functional groups were removed to yield 63k compounds. Docking with Glide SP (Schrödinger Suite 2020-2, Schrödinger, LLC, New York, NY, 2020) was performed as described below, and after clustering, from the top 500 hits, 13 diverse compounds were purchased. Second, the FKKTlib was filtered and all 9 and 13 compounds available in solid form with cyclobutenedione and thiourea core motifs, respectively, were experimentally evaluated.

### 2.4 LBVS With LiSiCA Software

Ligand-based virtual screening (LBVS) of the ZINC database of purchasable compounds using LiSiCA software (Lešnik et al., 2015) was performed with the bioactive 3D conformation of **cmp2105** as the reference compound (PDB ID: 6QZH, ligand **JLW**) (Jaeger et al., 2019). The double bonds of the thiadiazole-dioxide of the **JLW** ligand were correctly assigned, since they are missing in the PDB structure. Both 2D and 3D options of the LiSiCA were used with all other settings set to default values. From the 200 compounds most similar to the reference **cmp2105** according to the Tanimoto score, 27 diverse compounds were purchased—12 of them arising from the 2D method and 15 were discovered with the 3D method.

### 2.5 LBVS With ROCS Software

The MolPort library was screened using ROCS 3.3.2.2 software (OpenEye Scientific Software, Inc, Santa Fe, NM, United States; www.eyesopen.com) (Hawkins et al., 2007). For model A, a 3D pose for the first query, **navarixin**, was obtained by docking with Glide XP to the prepared CCR7 receptor, as described below. The bioactive 3D conformation of **cmp2105** (PDB ID: 6QZH, ligand **JLW**) (Jaeger et al., 2019) was used to create models B and C. All models were validated with the set of active compounds and generated decoys. The default settings of ROCS were used for virtual screening of all three queries. Virtual hits were prioritized based on the ComboScore, which considers similarity of 3D shape (“ShapeTanimoto”) and chemical pattern (“ColorScore”). For each query, 27–30 top scoring compounds were purchased from the clustered list of top 100 scoring hits.

### 2.6 Homology Modelling

Before the release of the CCR7 X-ray crystal, a homology model of CCR7 was built using the structure of human CCR9 (PDB ID: 5LWE; B chain). The template was identified by running 10 PSI Blast iterations on the starting CCR7 (UniProt ID: P32248) sequence to identify five top scoring templates (PDB IDs: 5LWE, 5UIW, 4YAY, 5WB2 and 5UNF) (Müller et al., 1999). The alignment and template was used to build the homology model using YASARA Twinset software (Krieger et al., 2002; Krieger and Vriend, 2015) using the following parameters: speed: Slow, EValue Max: 0.5, Templates Total: 5, Templates SameSeq: OligoState: 4, Alignments: 5, LoopSamples: 50 and TermExtension: 10.18 models were built and each model subjected to an unrestrained energy minimization with explicit water molecules by simulated annealing employing the YASARA2 force field (Krieger et al., 2009). The models were rated according to a quality Z-score and the best scoring model was used. The latter contained 276 of 378 target residues (73.0%) aligned to template residues. The sequence identity was 46.0% and the sequence similarity 68.1% (BLOSUM62 > 0). The monomer homology model after full unrestrained simulated annealing minimization was rated as optimal by YASARA with internal quality Z-score of 0.110, comprised amino acids 47-352, and was further checked with WHAT-IF test set.

### 2.7 Receptor Preparation

The CCR7 receptor was prepared from the X-ray crystal structure (PDB ID: 6QZH) (Jaeger et al., 2019) using Protein Preparation Wizard (Schrödinger Suite 2020-2, Schrödinger, LLC, New York, NY, 2020) (Madhavi Sastry et al., 2013). Briefly, missing side chains and missing loop 255-261 were modelled with Prime (Jacobson et al., 2004), hydrogen atoms were added, residues were protonated at pH 7.0, the hydrogen bonding network was refined, waters beyond 3.0 Å from other heteroatoms were removed, and restrained minimization was performed. The double bonds of the thiadiazole-dioxide core in the co-crystalized ligand **cmp2105** (PDB ID: 6QZH, ligand **JLW)** were assigned (Jaeger et al., 2019). Only the allosteric binding site was considered for pharmacophore-based screening and molecular docking.

### 2.8 Pharmacophore-Based Screening

The prepared CCR7 receptor was used to generate a structure-based pharmacophore model using LigandScout 4.4 (Inte:Ligand GmbH) (Wolber and Langer, 2005). Exclusion volumes defining regions based on the shape of the binding site residues were generated, and all features were converted to vectors. One hydrogen bond donor and one hydrophobic feature were marked as optional. This model was validated with the set of active compounds and generated decoys. Default settings in LigandScout were used. Virtual screening of the diversity set of compounds available from trusted commercial vendors yielded 78 virtual screening hits, which were then visually inspected, clustered according to Morgan fingerprints, and 23 diverse compounds were purchased.

### 2.9 Molecular Docking With FRED and Glide Software

Molecular docking was performed sequentially with FRED 3.4.0.2 (OpenEye Scientific Software, Inc, Santa Fe, NM, United States; www.eyesopen.com) and Glide software (Schrödinger Suite 2020-2: Glide, Schrödinger, LLC, New York, NY, 2020). First, Make Receptor 3.4.0.2 (OpenEye Scientific Software, Inc, Santa Fe, NM, United States; www.eyesopen.com) was used to define grid box of the allosteric binding site of the prepared receptor. The volume of the box was 6,725 Å^3^ (17.75 Å × 21.15 Å × 17.92 Å) and size of the outer contour was reduced to 1,139 Å^3^. Re-docking of the co-crystalized ligand **cmp2105** using FRED and Glide SP resulted in a root-mean-square deviation (RMSD) of 0.77 Å and 0.34 Å, respectively, confirming the validity of the pose prediction during docking. In the same manner, docking with Glide XP (Schrödinger Suite 2020-2, Schrödinger, LLC, New York, NY, 2020) (Friesner et al., 2006) was used to obtain the bioactive 3D conformation of **navarixin**. The MolPort library was docked with FRED and 100,000 highest scoring hits were used for sequential docking with Glide. A 3D structure of one stereoisomer was generated using LigPrep (Schrödinger Suite 2020-2, Schrödinger, LLC, New York, NY, 2020). The prepared receptor’s grid box was centered on the co-crystallized ligand and docking was performed using Glide SP (Schrödinger Suite 2020-2, Schrödinger, LLC, New York, NY, 2020) (Friesner et al., 2004). The 100 highest scoring virtual hits were clustered according to Morgan fingerprints, and 46 diverse compounds were purchased.

### 2.10 Molecular Docking With ProBiS-Dock Algorithm

Molecular docking with ProBiS-Dock algorithm (Konc et al., 2022) was performed with the prepared CCR7 receptor. Similar receptors with allosterically bound ligands that were known at the time of screening, i.e., CCR9 (PDB ID: 5LWE, ligand **79K** [**vercirnon**]) (Oswald et al., 2016) and CCR2 (PDB ID: 5T1A, ligand **VT5**) (Zheng et al., 2016), were aligned to the prepared CCR7 receptor. All three ligands, namely **cmp2105**, **vercirnon** and **VT5**, were extracted and used as template ligands that are required for molecular docking with ProBiS-Dock. Re-docking of the co-crystalized ligand **cmp2105** with an RMSD of 0.77 Å was performed for validation. From the MolPort library, one million compounds were randomly selected and used for virtual screening. The top 450 virtual hits were clustered according to Morgan fingerprints, and 40 diverse compounds were purchased.

### 2.11 Coupled Virtual Screening Approach

For the LBVS coupled to SBVS approach, we examined similar protein complexes published in the PDB database using ProBiS server (https://probis.nih.gov/) to identify all possible binding sites and small-molecule binding modes on the CCR7. Therefore, the built CCR7 homology model was used as an input for ProBiS calculation and one binding site identified (*binding site one in ProBiS; proximity of ligand **vercirnon** from CCR9 PDB ID: 5LWE*) (Konc and Janežič, 2012). The postulated binding site comprised of two pockets was defined by residues: Thr93, Leu147, Ile150, Val264, Ile265, Val268 and Val79, Val80, Thr82, Tyr83, Phe86, Asp94, Thr95, Leu97, Leu98, Leu100, Asp110, Asp336. Model was later validated with an all-atom RMSD of 1.942 Å towards PDB 6QZH crystal with allosteric site correctly identified relative to crystal **JLW** ligand. With the binding site defined, the receptor structure was generated using OEDocking 3.2.0.2 software package (OpenEye Scientific Software, Inc, Santa Fe, NM, United States; www.eyesopen.com) with MakeReceptor. A box with the volume of 5,805 Å^3^ (18,33 × 19,00 × 16,67 Å) was defined around reference ligand **vercirnon**. A balanced site shape potential was calculated where docking volume was 587 Å^3^. No constraints were used. Docking of the chemokine receptor targeted library (539,814 compounds) to the prepared receptor was performed using FRED from OpenEye as described above with dock_resolution parameter set to *High*. Top 100 scoring compounds according to Chemgauss4 score were collected, clustered according to Morgan fingerprints (20 clusters, average RMSD linkage) and best scoring representatives selected for purchase and testing.

## 3 Results and Discussion

### 3.1 Experimental Hit Validation

**FIGURE 2:**
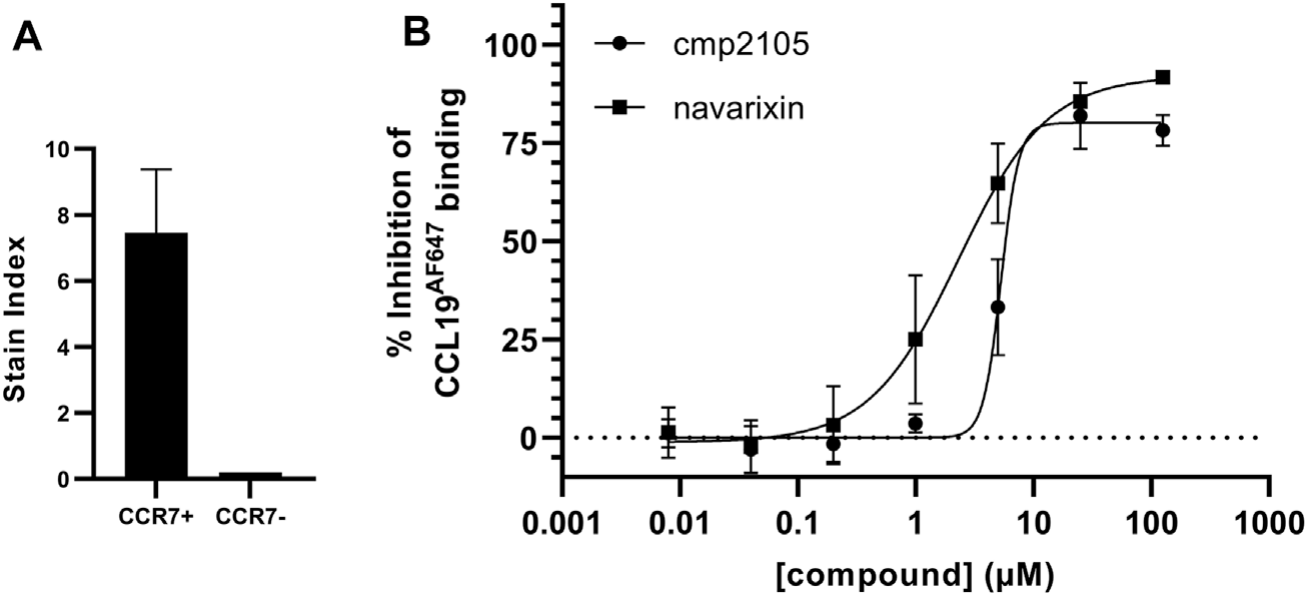
Inhibition of CCL19^AF647^ binding by **cmp2105** and **navarixin (A)** Incubation of U87 cells that overexpress CCR7 (CCR7+) with CCL19^AF647^ generates a strong fluorescent binding signal, which is not present when CCL19^AF647^ is incubated with cells that do not overexpress CCR7 (CCR7-) (Mean stain index ±SD of two (CCR7-) or four (CCR7+) independent experiments) **(B)** Dose dependent inhibition of CCL19^AF647^ binding by **cmp2105** and **navarixin**.

Before we initiated an extensive *in silico*-based screening program, several compounds described in the literature were either resynthesized (see Supplementary material) or purchased from commercial vendors to confirm their CCR7 antagonism in various pharmacological assays. Compounds previously shown to be CCR7 antagonists (i.e., **cmp2105**, **navarixin**, **CS-2**, and **CS-3**), as well as inactive control compounds (**maraviroc** and **vercirnon**), were included in this study (Figure 1; Table 1). A competition binding assay was established based on the specific interaction of fluorescently labeled CCL19 with CCR7 overexpressed on whole living cells (Figure 2A). Using CCL19^AF647^ as a tracer, the binding affinity for **cmp2105**, **navarixin**, **CS-2** and **CS-3** was evaluated (Figure 2B; Table 1). Whereas dose-dependent inhibition of CCL19^AF647^ binding was confirmed for **cmp2105** and **navarixin**, **CS-2** and **CS-3** did not show any CCR7 affinity. The previously observed stabilizing effect of CS-2 and CS-3 in thermal stability experiments was much smaller than for **cmp2105** and **navarixin** (Jaeger et al., 2019), suggesting a very low binding affinity for CCR7. It should further be noted that for **cmp2105** an IC_50_ value of 35 nM was previously reported when this compound was assessed in membrane-based competition experiments using radioactively labeled CCL19 (Jaeger et al., 2019). The fact that in our assay whole cells are used instead of membrane preparation, which requires the compound to first enter the cell before reaching its intracellular binding pocket, may therefore partly explain the increased apparent IC_50_ value observed here.

**TABLE 1:** CCL19 competition binding, CCR7 calcium mobilization and CCR7 β-arrestin data of reference compounds.

Compound	β-arrestin IC_50_ (µM)^a^	Calcium assay IC_50_ (µM)^b^	Binding assay IC_50_ (µM)^c^
Cmp2105	7.3	7.30 ± 1.66	6.12 ± 2.36
Navarixin	33.9	17.39 ± 1.12	2.43 ± 0.98
CS-2	NA^d^	>50 µM	>50 µM
CS-3	NA^d^	>50 µM	>50 µM
Vercirnon	NA^d^	>50 µM	ND^e^
Maraviroc	NA^d^	>50 µM	ND^e^

a IC_50_: compound concentration inhibiting β-arrestin recruitment in CHO-K1 cells by 50%. Data from (Jaeger et al., 2019).

b IC_50_: compound concentration inhibiting CCL19 induced intracellular calcium flux by 50% (Mean ± SD of at least three independent experiments).

c IC_50_: compound concentration inhibiting CCL19^AF647^binding by 50% (Mean ± SD of four independent experiments).

d NA: not available.

e ND: not determined.

**FIGURE 3:**
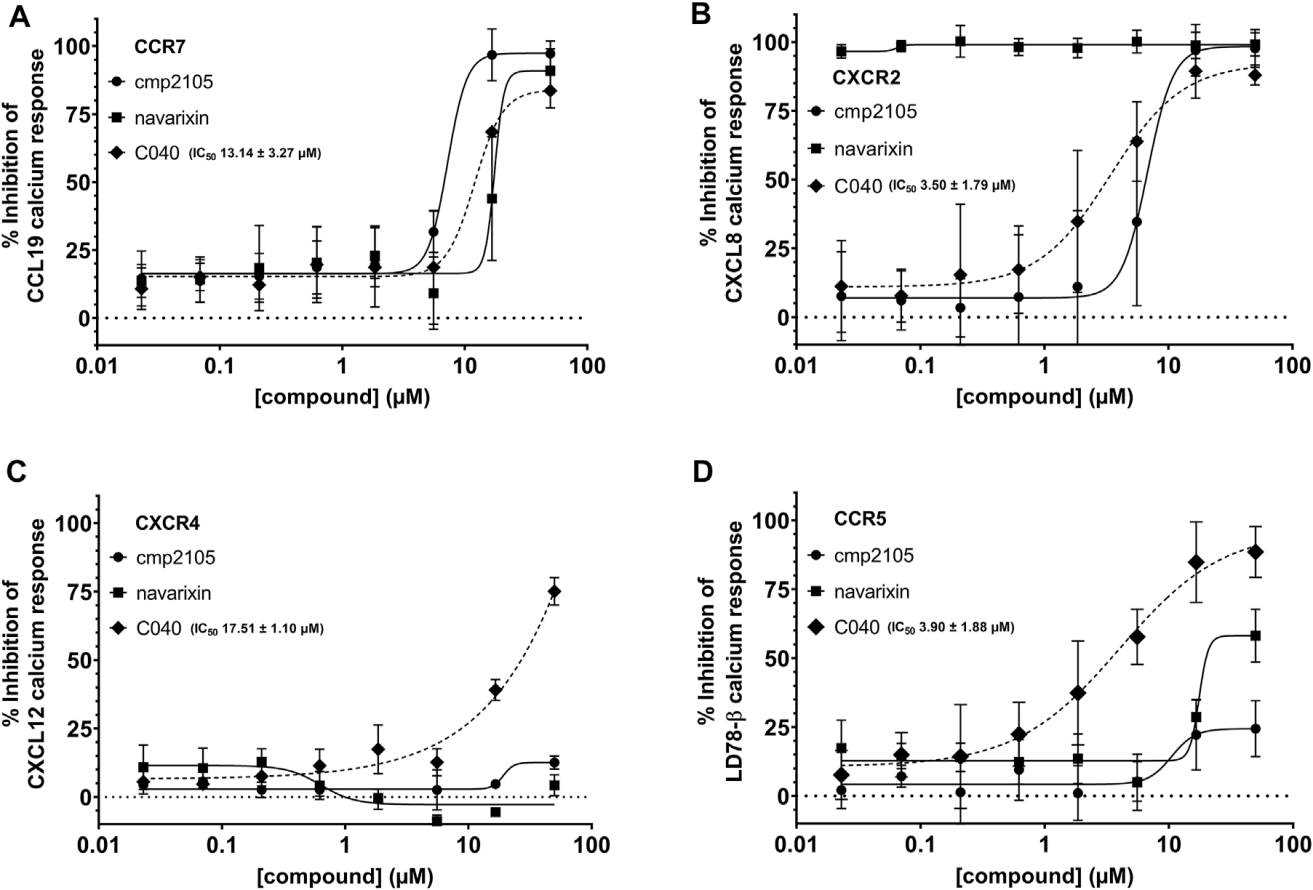
Inhibition of the intracellular Ca^2+^ release. The ability of **cmp2105**, **navarixin**, and **C040** to inhibit the Ca^2+^ response induced by **(A)** CCL19-CCR7 **(B)** CXCL8-CXCR2 **(C)** CXCL12-CXCR4, and **(D)** LD78-β-CCR5 was evaluated. Mean ± SD of at least three independent experiments is shown.

Reference compounds were also evaluated in a CCR7 kinetic, fluorescence-based calcium mobilization assay. **Cmp2105** and **navarixin** showed IC_50_ values in the 5–15 µM range for antagonizing the CCL19-induced calcium response (Figure 3; Table 1) in line with their CCR7 antagonistic activity previously determined in a β-arrestin recruitment assay (Jaeger et al., 2019). In agreement with the lack of observed binding affinity, **CS-2** and **CS-3** were also inactive in this CCR7 calcium mobilization assay (Table 1). Furthermore, the absence of activity of **vercirnon** and **maraviroc** in the calcium mobilization assay is in agreement with their lack of activity in the thermal shift assay (Jaeger et al., 2019).

### 3.2 Virtual Screening Campaign

**FIGURE 4:**
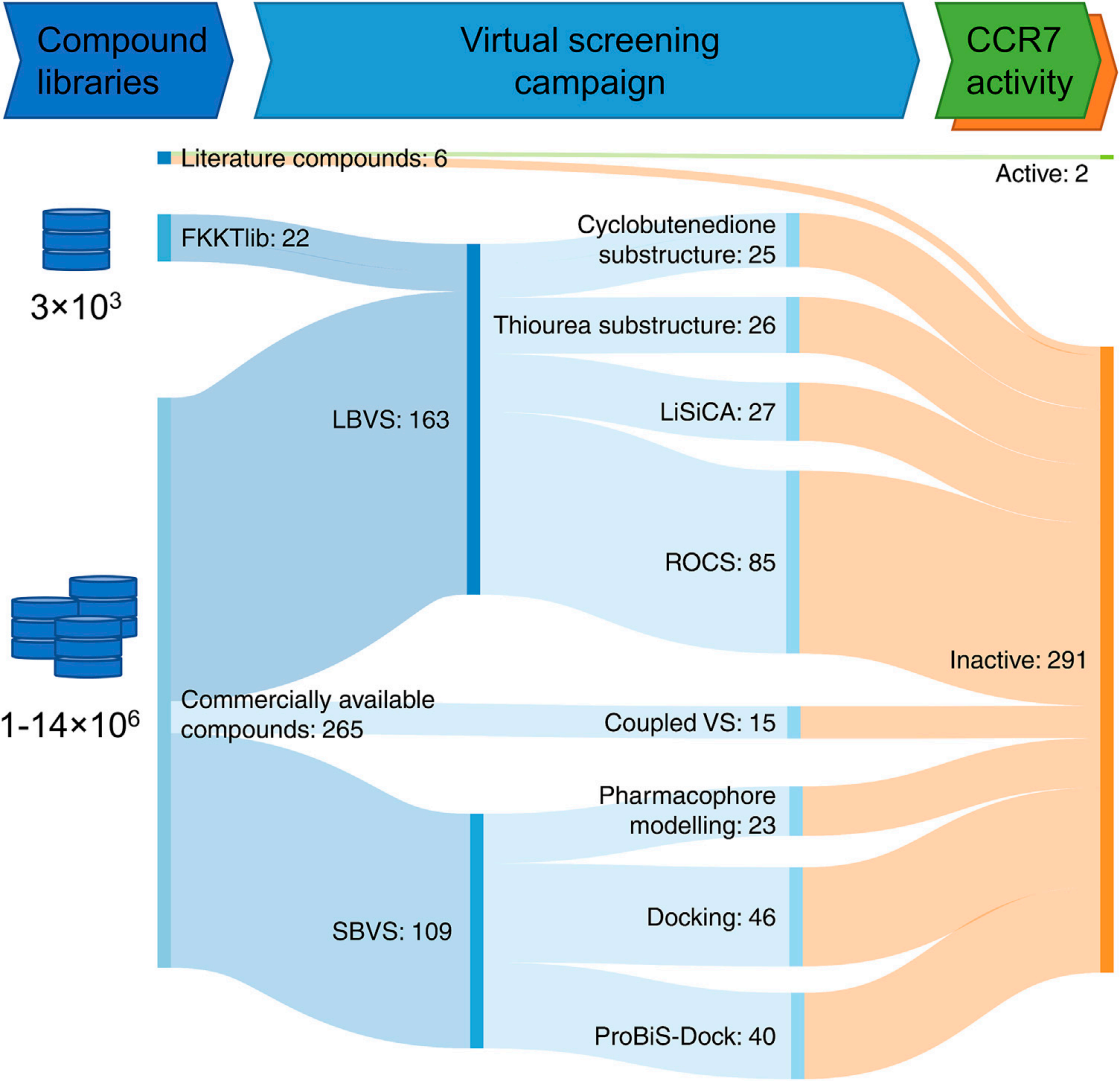
Virtual screening workflow represented as a Sankey diagram. Compounds described in this manuscript were obtained from the scientific literature, the FKKTlib academic library, and databases of commercially available compounds. Different ligand-based virtual screening (LBVS) (core motif substructure search, screening with LiSiCA and ROCS software), structure-based virtual screening (SBVS) (pharmacophore screening and docking) and coupled virtual screening (VS.) approaches were explored. Altogether 293 compounds were tested in the CCL19 induced calcium signaling CCR7 assay.

To expand the current set of potent CCR7 modulators, we launched a virtual screening campaign. Based on known CCR7 ligands, in particular **cmp2105** and **navarixin**, an LBVS was performed. Furthermore, a recently published crystal structure of the receptor (Jaeger et al., 2019) was used for structure-based virtual screening (SBVS). In addition to libraries of commercially available compounds, we also used the FKKTlib academic library for screening (Figure 4).

LBVS is commonly used in drug discovery and is based on the assumption that structurally similar compounds have similar biological properties. Various metrics are used to express similarity between compounds (Maggiora et al., 2014). We started with a simple substructure search for core motifs that are typical for small molecule ligands targeting the intracellular binding sites of various chemokine receptors. Thioureas bind to an intracellular binding pocket of CXCR2 (Nicholls et al., 2008) and cyclobutenediones have been shown to bind intracellularly to CXCR2 (Liu et al., 2021, 2) and CCR7 (Jaeger et al., 2019). This approach was applied to both the FKKTlib academic library and a library of commercially available compounds.

Second, we used ligand-based virtual screening software LiSiCA (Lešnik et al., 2015) to find compounds with different scaffolds and core motifs than those of the reference compound **cmp2105**. LiSiCA is based on a graph-theoretical representation of molecules and uses a fast maximum clique algorithm (Konc and Janežič, 2007) to search for 2D or 3D similarities between a reference compound and a database of target compounds. The similarities found are expressed by the Tanimoto coefficients. A library of commercially available compounds was compared to the reference based on both 2D and 3D molecular representations.

**FIGURE 5:**
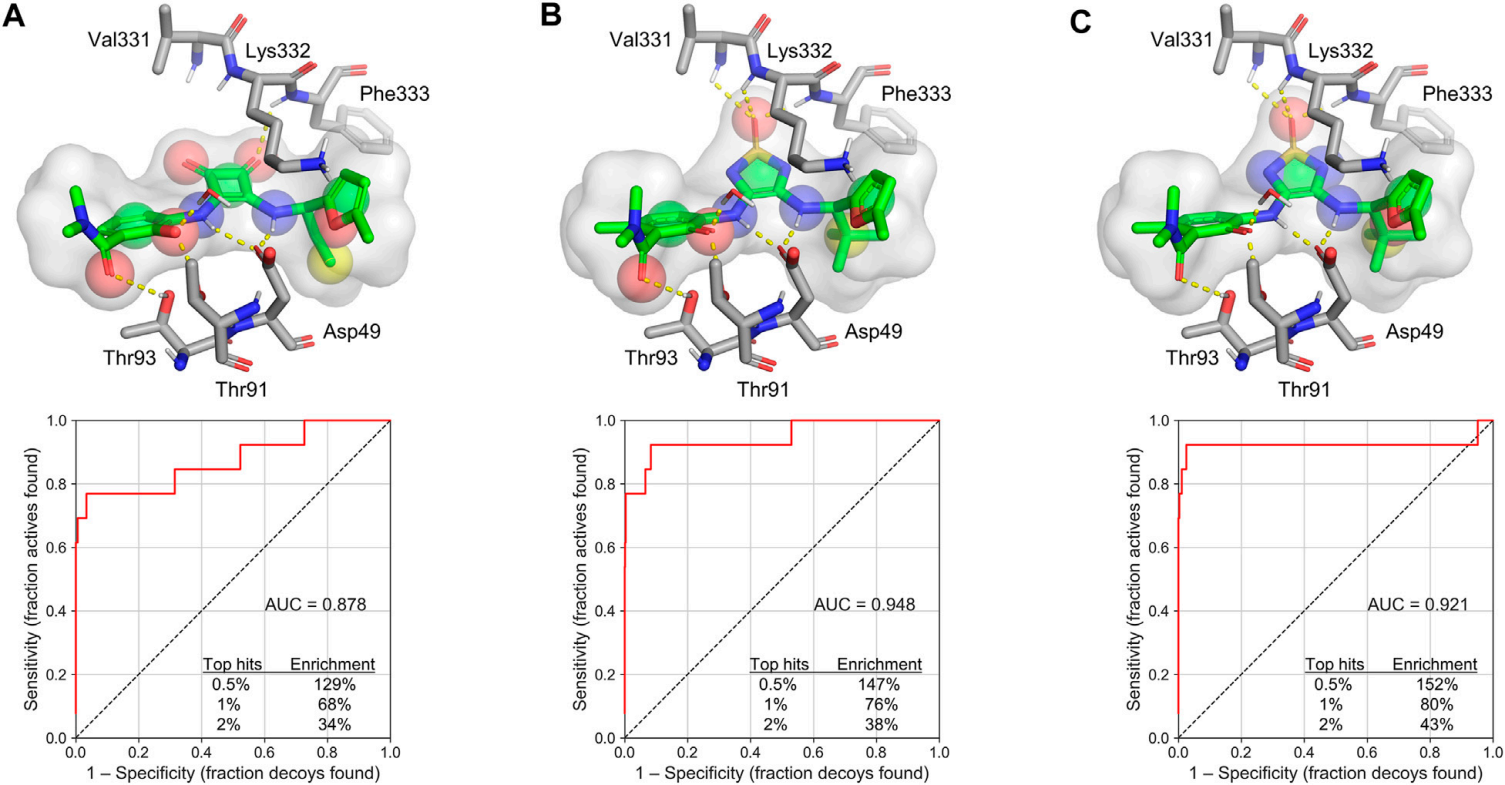
ROCS models and their corresponding ROC plots **(A) Navarixin (B) cmp2105** with relevant color features as determined from the crystal structure, and **(C) cmp2105** with color features extending into the inner part of the binding pocket. The color features are shown as spheres: hydrogen bond acceptor (red spheres), hydrogen bond donor (blue spheres), hydrophobic region (yellow spheres), and ring (green spheres). AUC = area under the curve.

Third, 3D shape-based virtual screening was performed by rapid overlay of chemical structures (ROCS) (Hawkins et al., 2007). This method is based on the concept that compounds have a similar shape if their volumes, described by a Gaussian function, overlap well. In addition to molecular volume, a color force field is used to describe other molecular features, such as hydrogen bond donors and acceptors, anions, cations, hydrophobes and rings (Kirchmair et al., 2009). As a starting point for the ROCS search, we modeled a 3D conformation of **navarixin** by docking with Glide XP and used it to generate ROCS model A (Figure 5A). The bioactive conformation of **cmp2105** was extracted from the co-crystal structure (PDB ID: 6QZH) and used directly to create models B and C, which differed in the selection of color features. Only relevant hydrogen bond donors and acceptors based on the distances in the crystal structure were used for the model B (Figure 5B). For model C, only color features in the inner part of the binding pocket were selected, leaving more degrees of freedom for the part of the molecule that extends toward the solvent (Figure 5C). All three models performed well in screening a set of active compounds and generated decoys. The results are presented in the form of receiver-operating characteristic (ROC) curves (Figure 5). Subsequently, the models were used to screen a library of commercially available compounds.

**FIGURE 6:**
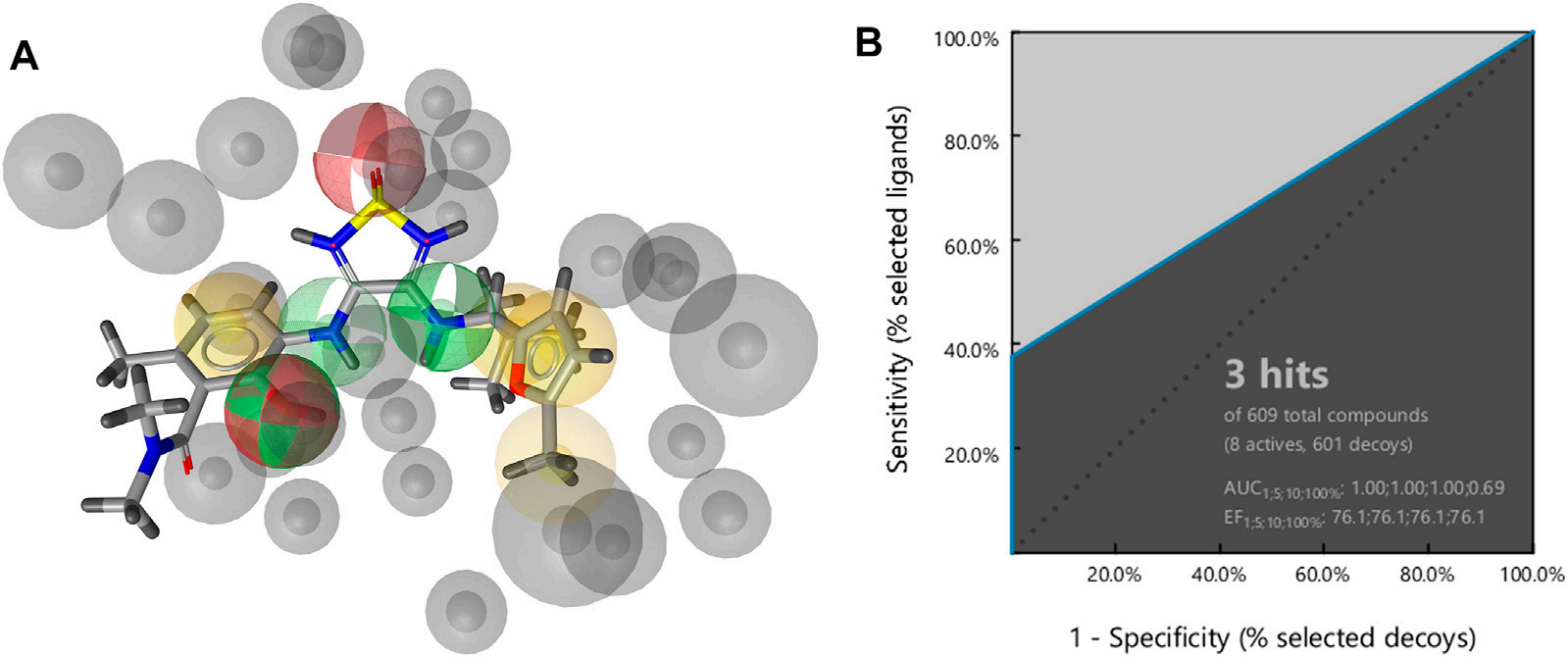
**(A)** Structure-based pharmacophore model with four hydrophobic features (yellow spheres), two hydrogen bond acceptors (red spheres), three hydrogen bond donors (green spheres), and exclusion volumes (gray spheres) defining restricted regions based on the shape of the binding site residues. Optional features are marked as dashed **(B)** Resulting ROC plot from virtual screening of 609 compounds (8 active compounds and 601 generated decoys). TP = true positives; FP = false positives; AUC = area under the curve; EF = enrichment factor.

A structure-based pharmacophore model was constructed from the crystal structure of **cmp2105** (Figure 6A). The model consisted of a hydrogen bond acceptor for the sulfonyl moiety, two hydrogen bond donors for the secondary amines (one labeled as optional), a hydrogen bond donor and acceptor for the phenol moiety, four hydrophobic features, and exclusion spheres. A hydrophobic feature for the methyl moiety on furan ring was also labeled as optional. The results of screening a set of active compounds and generated decoys were visualized by ROC plot, with the rate of active compounds on the *y*-axis and the rate of decoys on the *x*-axis (Figure 6B). Three of eight active compounds were detected by this model, which was in turn used to screen a library of commercially available compounds.

**FIGURE 7:**
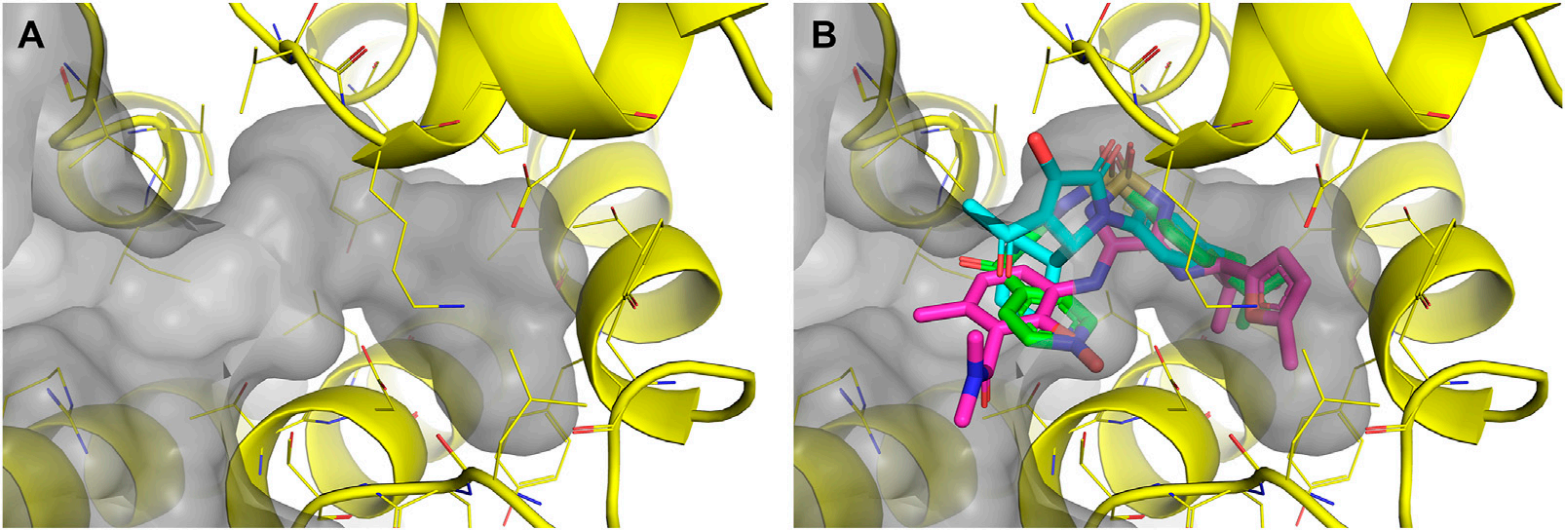
**(A)** The prepared CCR7 receptor (PDB ID: 6QZH, yellow) was used for docking with FRED and subsequently with Glide. The surface of the allosteric pocket is shown in gray **(B) **The input for the ProBiS-Dock algorithm consisted of the prepared CCR7 receptor (PDB ID: 6QZH, yellow) and three template ligands, **cmp2105** (magenta), **vercirnon** (green), **VT5** (cyan).

In the next SBVS approach, molecular docking into the allosteric binding site of CCR7 was employed (Figure 7A). First, we used FRED software (McGann, 2012), which was capable of high-throughput docking of a prepared library containing more than 3.5 million commercially available compounds. Then, only 100,000 highest-scoring compounds were used for subsequent docking with Glide software (Friesner et al., 2004), which was expected to be more successful in enriching a virtual hit list but is also more computationally intensive (Kellenberger et al., 2004; McGaughey et al., 2007). Besides, Glide performed better in a re-docking experiment with **cmp2105**, achieving an RMSD of 0.34 Å, compared to FRED with an RMSD of 0.77 Å.

A SBVS approach was also explored using the ProBiS-Dock algorithm (Konc et al., 2022). Allosteric binding sites of other chemokine receptor crystal structures available at the time of our study were aligned and compared. Accordingly, three template ligands were selected: **cmp2105** (CCR7), **vercirnon** (CCR9), and **VT5** (CCR2). The template ligands were used together with the CCR7 crystal structure (PDB ID: 6QZH) (Jaeger et al., 2019) as input to the ProBiS-Dock algorithm (Figure 7B). When docking the library of commercially available compounds, both the docked compound and the receptor were treated as fully flexible to account for the induced fit of ligand binding. The obtained poses were scored using a combination of a site-specific and a generalized statistical scoring function. A site-specific scoring function scores the docked compounds based on their overlap with the template ligands, while a generalized statistical scoring function scores the compounds based on their interactions with the receptor.

Finally, a coupled virtual screening approach was explored, in which a chemokine receptor targeted compound library containing 539,814 compounds was docked using FRED software. This library covered similar compounds to the literature actives on all chemokine receptors. The allosteric binding site of a CCR7 homology model, 5 Å around the **vercirnon** ligand from the template structure of CCR9, was used for docking.

**FIGURE 8:**
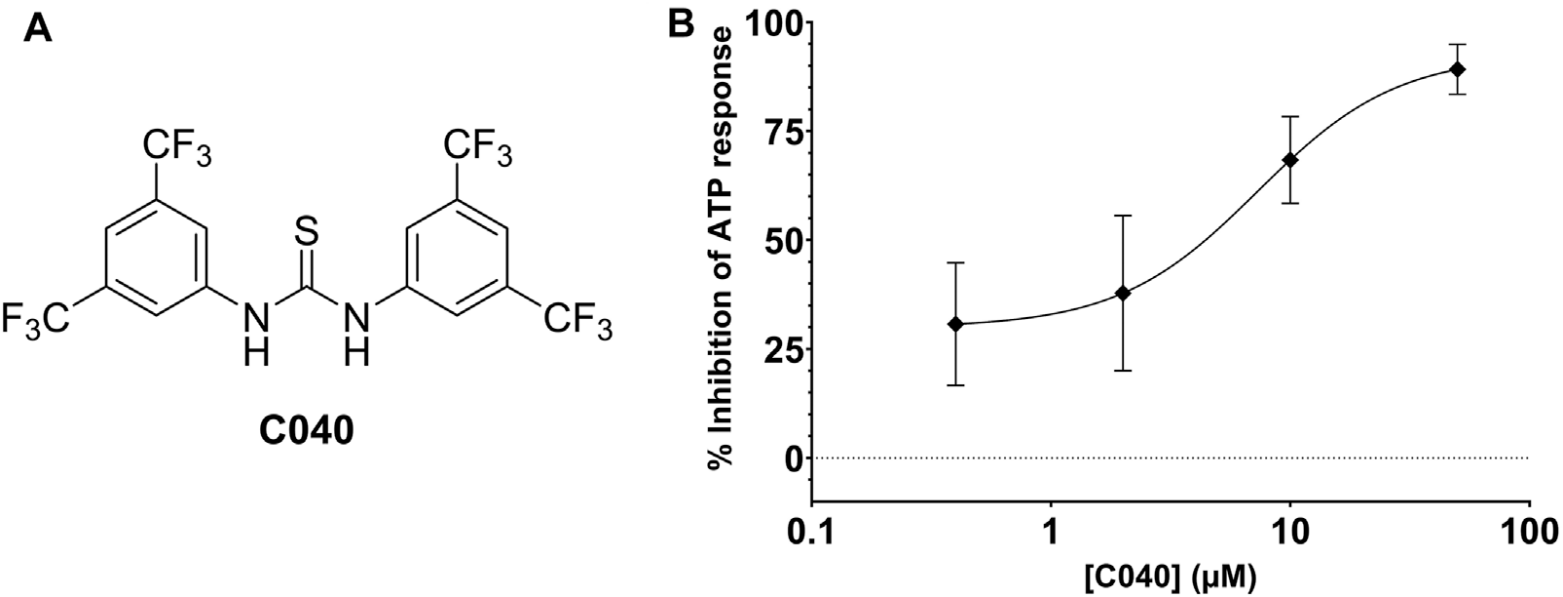
**(A)** Chemical structure of **C040** **(B)** Inhibition of the ATP-induced Ca^2+^ release by **C040**.

In total, 287 virtual screening hits were selected from the various approaches and experimentally evaluated as potential CCR7 antagonists in the calcium mobilization assay (Supplementary Excel File). One compound (**C040**, Figure 8A) showed dose-dependent inhibition of the CCR7-mediated calcium response, affording an EC_50_ value of 13.14 µM (Figure 3A). Given the conserved nature of the intracellular binding pocket targeted by **cmp2105** and **navarixin** (Jaeger et al., 2019), the inhibiting effect of **C040**, alongside the reference compounds **cmp2105** and **navarixin**, on the intracellular calcium mobilization mediated by several other chemokine receptors was evaluated (Figure 3B–D). **Cmp2105** and **navarixin** inhibited the CCR7 and CXCR2 mediated calcium mobilization, in line with literature data (Gonsiorek et al., 2007; Jaeger et al., 2019), but had no (or only very limited) effect on CXCR4 and CCR5 mediated responses. In contrast, **C040** completely lacked receptor specificity as it inhibited the calcium mobilization downstream all tested chemokine receptors, with similar potencies (IC_50_ values in the 3–18 µM range). These data suggested that the chemokine receptor antagonistic activity of **C040** may, at least partially, be due to interference with the fluorescent assay readout. To further explore this hypothesis, the ability of **C040** to inhibit calcium responses not mediated by human chemokine receptors was investigated. It is known that stimulation of CHO-K1 cells with adenosine triphosphate (ATP) leads to a rapid release of Ca^2+^ from intracellular stores (Iredale and Hill, 1993). Using CHO-K1 cells, exactly the same experimental set-up as for the chemokine receptor expressing U87 cells was applied, essentially including the same fluorescent calcium dye (Fluo-2) for cell loading. Also in this experimental setup **C040** was able to dose-dependently inhibit the measured calcium response induced by ATP (10 µM final concentration) (Figure 8B), confirming its interference with this particular fluorescent readout. Furthermore, when **C040** was assessed in the CCR7 competition binding assay described above, it was inactive at the highest concentration tested (25 µM). Altogether, these data indicate that **C040**, despite showing activity in the CCR7 calcium assay, should not be selected as a hit compound, for a medicinal chemistry-based optimization campaign.

## 4 Conclusion

The CCR7 antagonistic activity of previously reported ligands (**cmp2105** and **navarixin**) was confirmed in two independent assays, namely a kinetic, fluorescence-based calcium mobilization CCR7 assay and a CCR7 competition binding assay. Starting from this, an *in silico* virtual screening campaign for the identification of novel CCR7 antagonists was carried out using several strategies. A library of commercially available compounds and an academic library FKKTlib (available at: https://knjiznica-spojin.fkkt.uni-lj.si/fkktlib/) were used to prepare the input libraries. LBVS, SBVS, and coupled virtual screenings were followed by experimental validation. A selection of 287 *in silico* hits was experimentally investigated for CCR7 antagonism. Initial data revealed that one analogue (**C040**) showed promising CCR7 antagonistic activity in the calcium mobilization assay. Unfortunately, **C040** was equally active against other chemokine receptors tested and was completely devoid of activity in a CCR7 binding assay. Since **C040** also behaves as an antagonist of a purinergic receptor, it strongly suggests that **C040** interferes with the assay read-out, rather than being a bona fide chemokine receptor antagonist. This study highlights the importance of experimental validation of virtual hits, using an array of orthogonal assays to confirm activity before nominating any hits. Since none of the compounds disclosed in this manuscript showed any CCR7 antagonistic activity, we report them as a large set of inactive compounds that can be used by the medicinal chemistry community as a set of experimentally validated decoys. We believe this will facilitate the identification and computational design of new CCR7 ligands in the future.

## Data Availability

The datasets presented in this study can be found in online repositories. The names of the repository/repositories and accession number(s) can be found in the article/Supplementary Material.

## Abbreviations

ATP, adenosine triphosphate; CCR7, CC Chemokine Receptor 7; GPCRs, G protein-coupled receptors; LBVS, ligand-based virtual screening; LiSiCA, ligand similarity using clique algorithm; ProBiS-Dock, protein binding sites docking; ROC, receiver-operating characteristic; ROCS, rapid overlay of chemical structures; RMSD, root-mean-square deviation; SBVS, structure-based virtual screening.
