# Supplementary material for: A Set of Experimentally Validated Decoys for the Human CC Chemokine Receptor 7 (CCR7) Obtained by Virtual Screening
Source: Front Pharmacol. 2022 Mar 18;13:855653. doi: 10.3389/fphar.2022.855653 (PMC8972196; doi:10.3389/fphar.2022.855653)
Supplement: Supplementary file 1 [file DataSheet1.docx]

# SUPPLEMENTARY MATERIAL

**A set of experimentally validated decoys for the human CC Chemokine Receptor 7 (CCR7) obtained by virtual screening**

**Matic Proj ^1^, Steven De Jonghe ^2^, Tom Van Loy ^2^, Marko Jukič ^3,4^, Anže Meden ^1^, Luka Ciber ^5^, Črtomir Podlipnik ^5^, Uroš Grošelj ^5^, Janez Konc ^6^, Dominique Schols ^2^, Stanislav Gobec ^1,*^**

^1^ University of Ljubljana, Faculty of Pharmacy, Department of Pharmaceutical Chemistry, Ljubljana, Slovenia

^2^ KU Leuven, Department of Microbiology, Immunology and Transplantation, Rega Institute for Medical Research, Laboratory of Virology and Chemotherapy, Herestraat 49, box 1043, 3000 Leuven, Belgium

^3^ University of Maribor, Faculty of Chemistry and Chemical Engineering, Laboratory of Physical Chemistry and Chemical Thermodynamics, Maribor, Slovenia.

^4^ University of Primorska, Faculty of Mathematics, Natural Sciences and Information Technologies, Koper, Slovenia.

^5^ University of Ljubljana, Faculty of Chemistry and Chemical Technology, Ljubljana, Slovenia

^6^ National Institute of Chemistry, Ljubljana, Slovenia

*** Correspondence:**Stanislav Gobec
[stanislav.gobec@ffa.uni-lj.si](mailto:stanislav.gobec@ffa.uni-lj.si)

**Table of contents**

[FILTER configuration file S2](#_Toc96077156)

[Supporting figures S3](#_Toc96077157)

[Supporting tables S4](#_Toc96077158)

[Chemistry – General Information S8](#_Toc96077159)

[Supplementary references S14](#_Toc96077160)

## FILTER configuration file

ELIMINATE_METALS Sc,Ti,V,Cr,Mn,Fe,Co,Ni,Cu,Zn,Y,Zr,Nb,Mo,Tc,Ru,Rh,Pd,Ag,Cd

ALLOWED_ELEMENTS H,C,N,O,F,P,S,Cl,Br,I

#-------------------------------------------------------------------

MIN_MOLWT 200 "Minimum molecular weight"

MAX_MOLWT 800 "Maximum molecular weight"

#-------------------------------------------------------------------

#Calculated LogP

MIN_XLOGP -4.0 "Minimum XLogP"

MAX_XLOGP 6.85 "Maximum XLogP"

#-------------------------------------------------------------------

AGGREGATORS true "Eliminate known aggregators"

PRED_AGG true "Eliminate predicted aggregators"

#-------------------------------------------------------------------

#functional groups which often eliminate compounds from consideration

RULE 0 aldehyde

RULE 0 acid_halide

RULE 0 peroxide

RULE 0 sulfonyl_halide

RULE 0 carbonate

RULE 0 isonitrile

RULE 0 isocyanate

RULE 0 isothiocyanate

RULE 0 acyl_cyanides

RULE 0 acylhydrazide

RULE 0 alphahalo_amine

RULE 0 alphahalo_ketone

RULE 0 anhydride

RULE 0 azide

RULE 0 aziridine

RULE 0 azocyanamides

RULE 0 oxaziridine

RULE 0 phosphoranes

RULE 0 dithioacetal

RULE 0 hydrazine

RULE 0 nitroso

RULE 0 triazine

RULE 0 disulfide

RULE 0 sulfinylthio

RULE 0 sulfonylnitrile

RULE 0 sulfinimine

RULE 0 fluorenylmethoxycarbonyl_Fmoc

RULE 0 HOBT_esters

RULE 0 trimethylsilyl_TMS

RULE 0 cation_C_Cl_I_P_or_S

RULE 0 oxygen_cation

RULE 0 lawesson_s_reagent

RULE 0 nonacylhydrazone

RULE 0 N_methoyl

RULE 0 N_P_S_Halides

RULE 0 NS_beta_halothyl

RULE 0 cytochalasin_derivatives

RULE 0 squalestatin_derivatives

RULE 0 monensin_derivatives

RULE 0 iodine

RULE 0 iodoso

RULE 0 iodoxy

## Supporting figures

**Figure S1.** Known CCR7 antagonists that were used as a set of active compounds in the virtual screening campaign (Taveras et al., 2010; Jaeger et al., 2019).

## Supporting tables

**Table S1.** Compounds active on multiple chemokine receptors (CCR1, CCR2, CCR3, CCR4, CCR5, CCR7, CCR8, CCR9, CCR10, CXCR1, CXCR2, CXCR3, CXCR4, and CXCR7), used to construct a chemokine receptor targeted compound library.

| 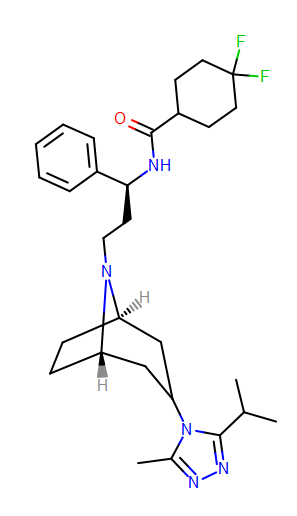  Maraviroc, CCR5 | 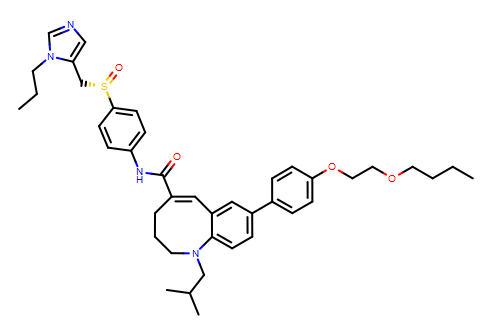  Cenicriviroc, CCR2, CCR5 | 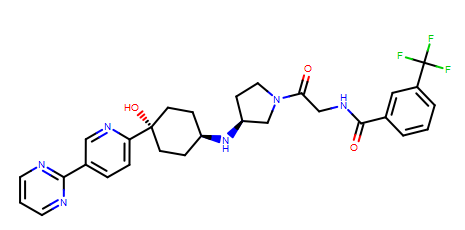  PF-4136309, CCR2 | 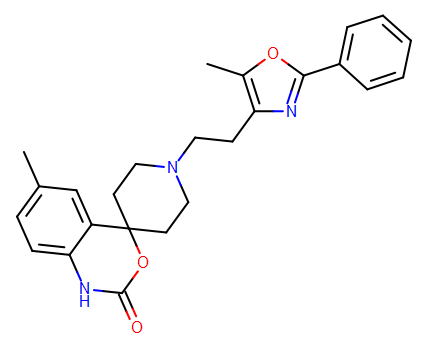  RS 504393, CCR2 |
| --- | --- | --- | --- |
| 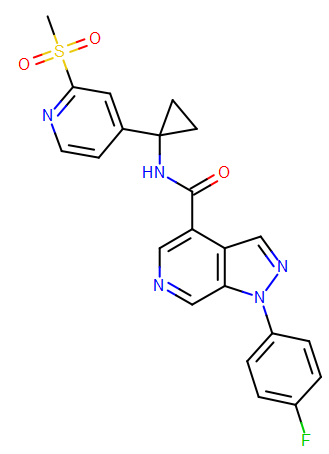  CCR1 antagonist 8, CCR1 | 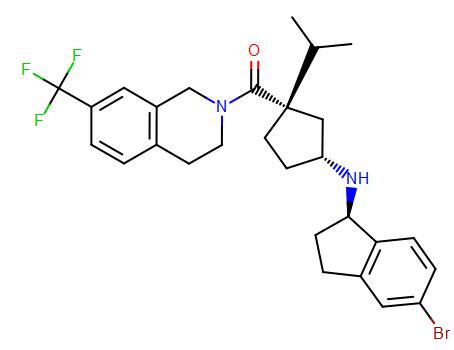  CCR2 antagonist 1, CCR2 | 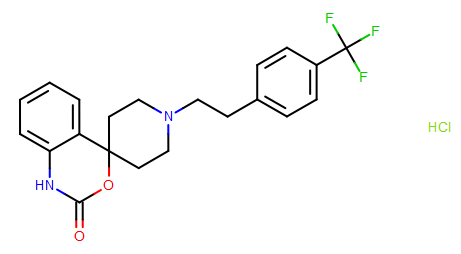  RS102895 hydrochloride, CCR1, CCR2 | 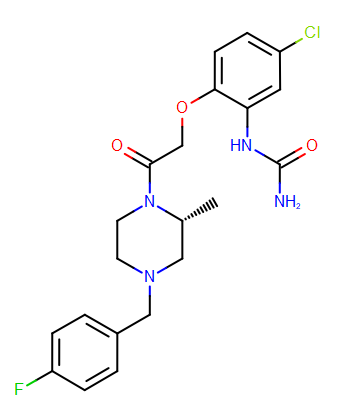  BX471, CCR1 |
| 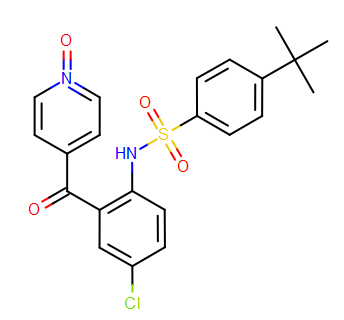  Vercirnon, CCR9 | 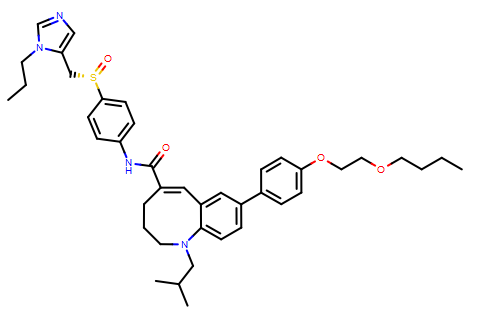  Cenicriviroc mesylate, CCR2, CCR5 | 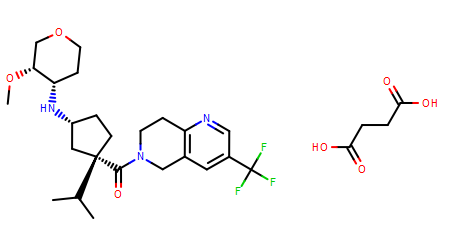  MK-0812 succinate, CCR2 | 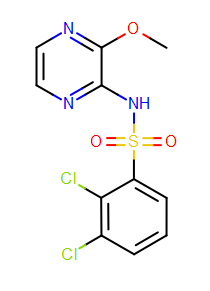  AZD2098, CCR4 |
| 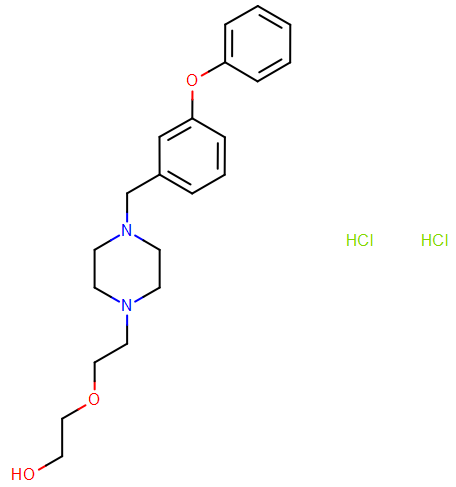  ZK756326 dihydrochloride, CCR8 | 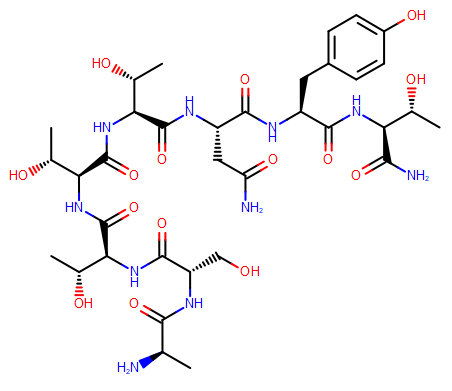  DAPTA, CCR5 | 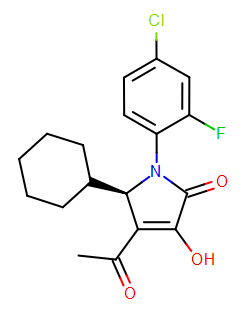  CCR2-RA-[R], CCR2 | 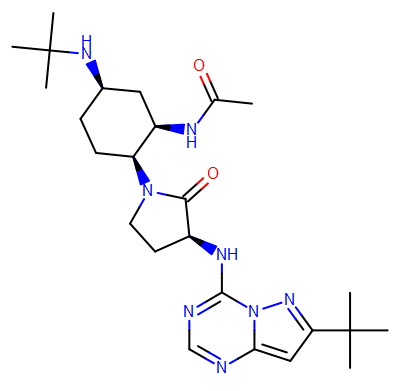  BMS-813160, CCR2, CCR5 |
| 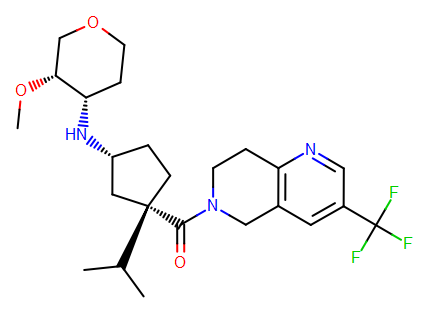  MK-0812, CCR2 | 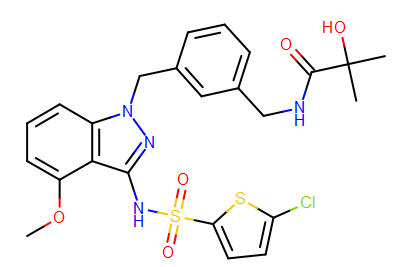  GSK2239633A, CCR4 | 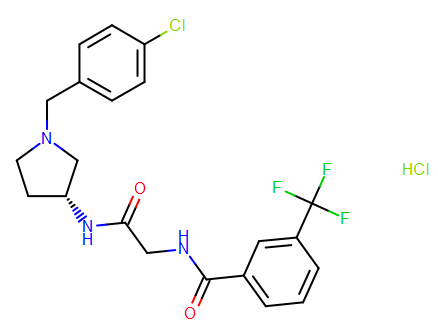  Teijin compound 1, CCR2 | 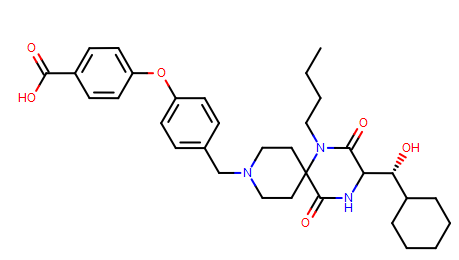  Aplaviroc, CCR5 |
| 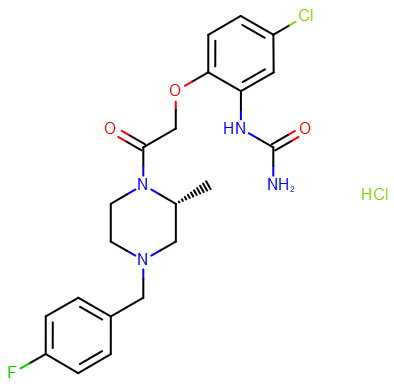  BX-471 hydrochloride, CCR1 | 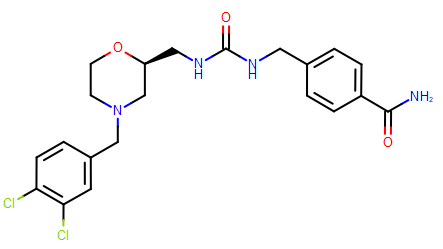  GW 766994, CCR3 | 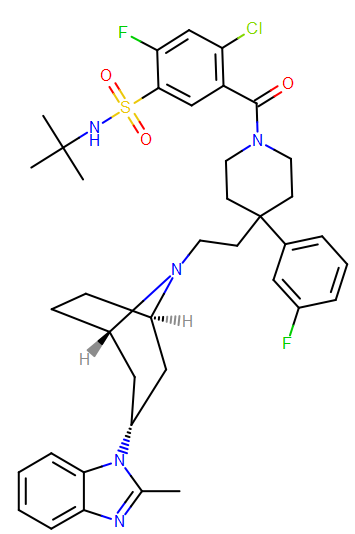  CCR5 antagonist 1, CCR5 | 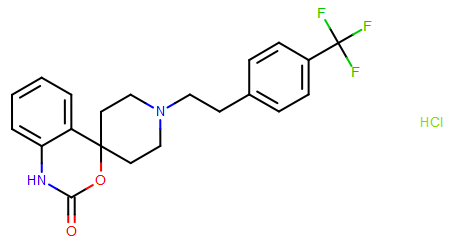  RS102895, CCR1, CCR2 |
| 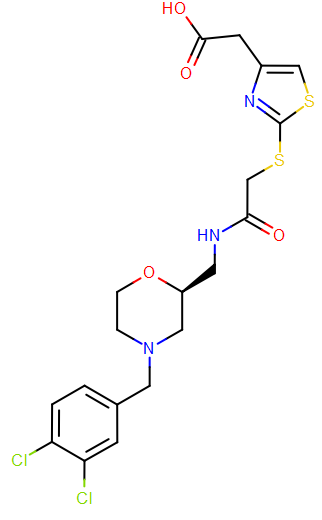  CCR3 antagonist 1, CCR3 | 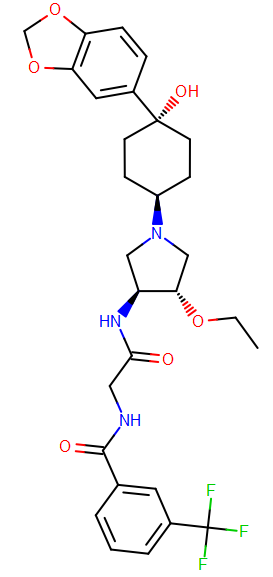  INCB3344, CCR2 | 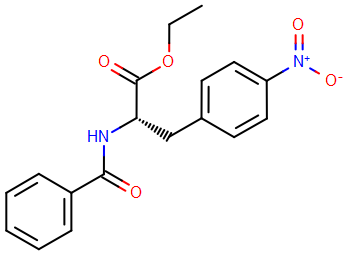  SB297006, CCR3 | 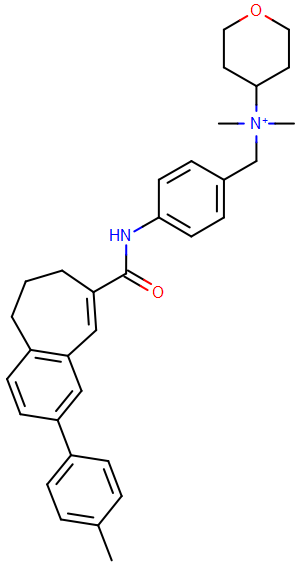  TAK-779, CCR3, CCR5, CXCR3 |
| 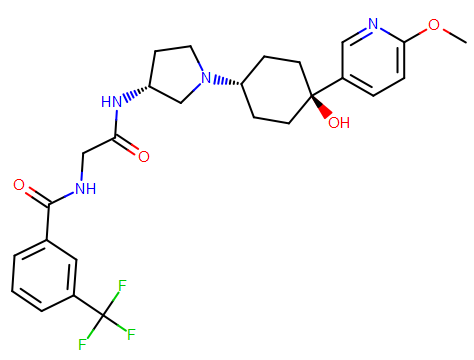  INCB 3284, CCR2 | 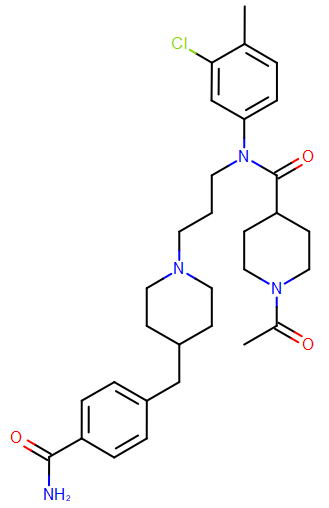  TAK-220, CCR5 | 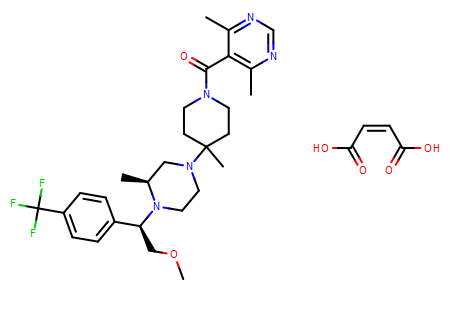  Vicriviroc maleate, CCR5 | 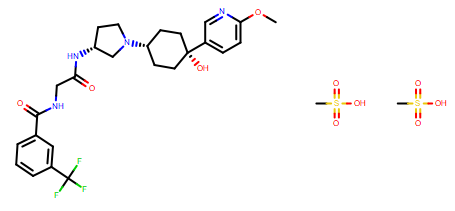  INCB 3284 dimesylate, CCR2 |
| 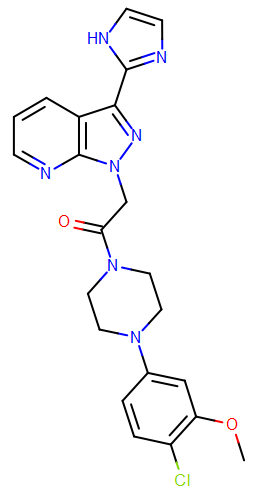  CCR1 antagonist 1, CCR1 | 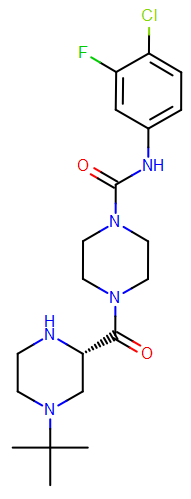  AZD2423, CCR2 | 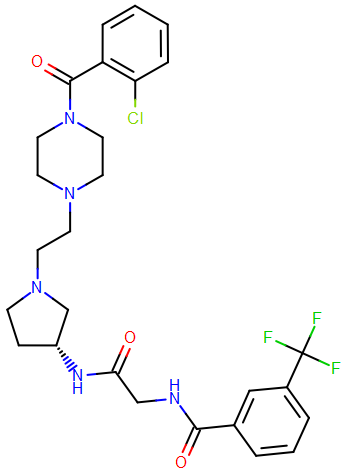  YJC-10592, CCR2 | 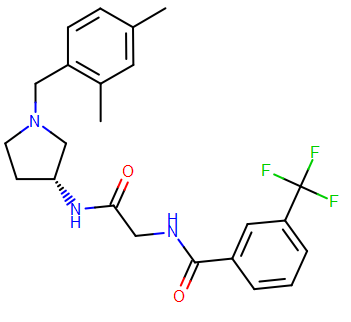  CCR251, CCR2 |
| 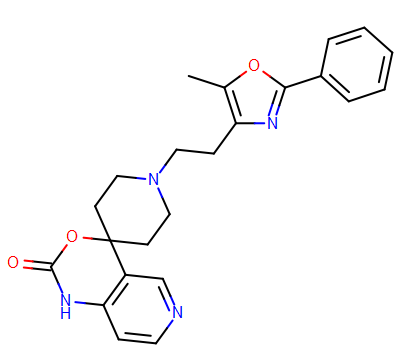  CCR252, CCR2 | 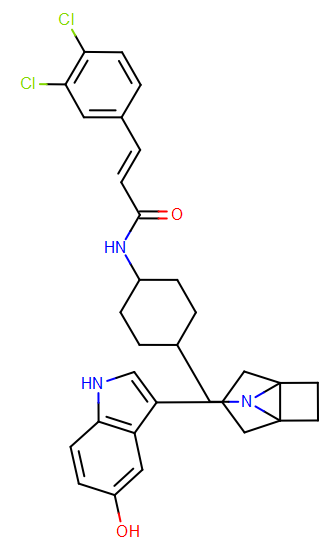  CCR253, CCR2 | 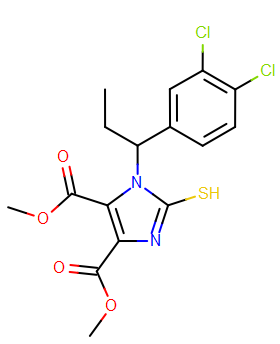  JNJ27553292, CCR2 | 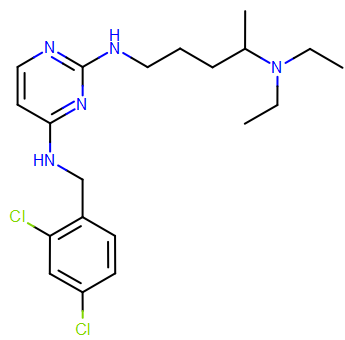  CCR4Ant1, CCR4 |
| 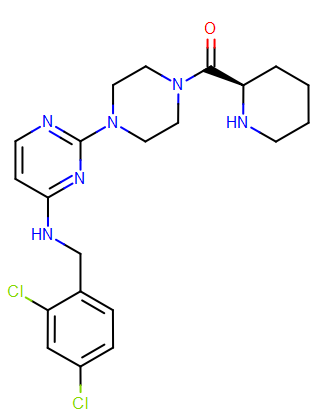  CCR4Ant2, CCR4 | 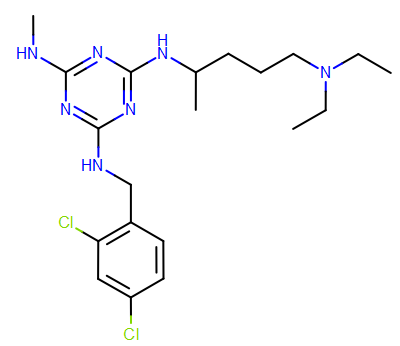  CCR4Ant3, CCR4 | 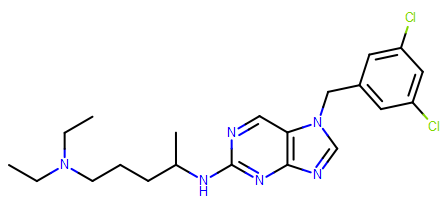  CCR4Ant11, CCR4 | 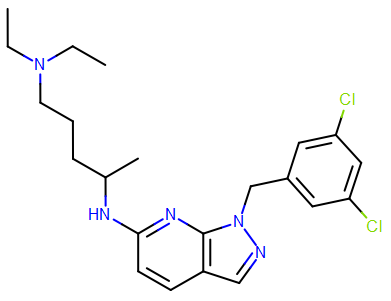  CCR4Ant15, CCR4 |
| 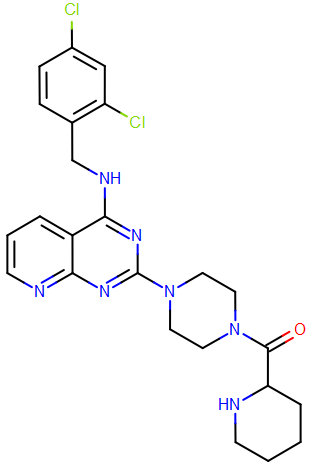  CCR4Ant22 | 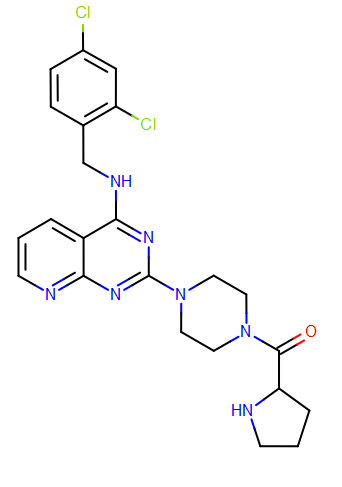  CCR4Ant23, CCR4 | 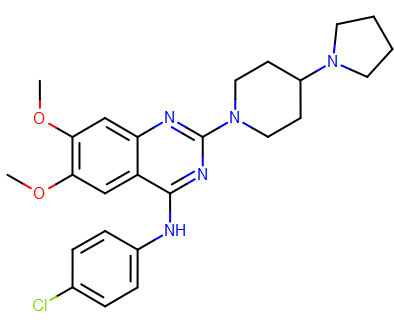  CCR4Ast, CCR4 | 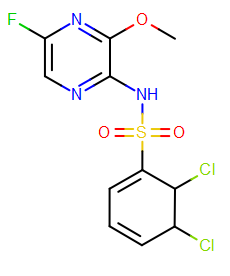  AZD1678, CCR4 |
| 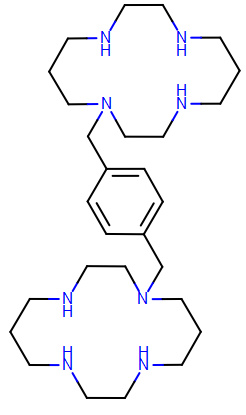  AMD3100 (Plerixafor), CCR7, CXCR4 | 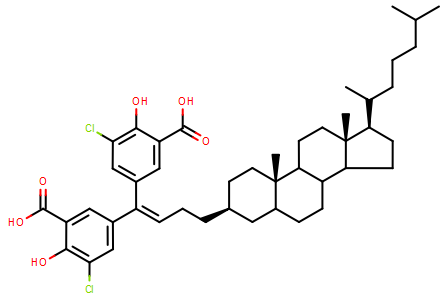  Cosalane, CCR7 | 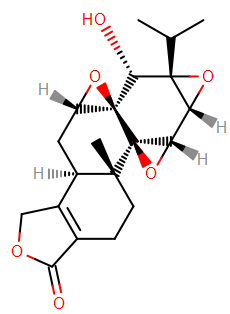  Triptolide, CCR7 | 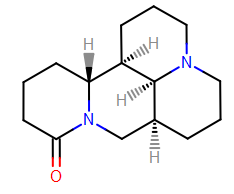  Matrine, CCR7 |
| 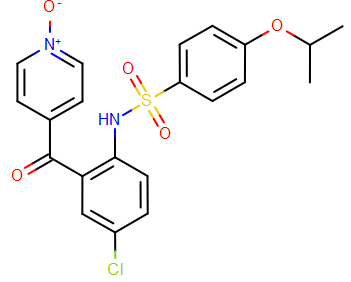  MLN3126, CCR9 | 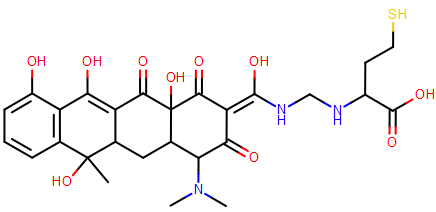  CCR9Ant31, CCR9 | 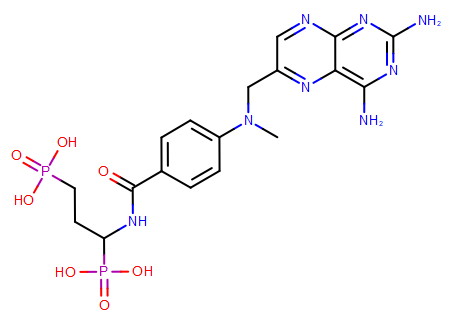  CCR9Ant32, CCR9 | 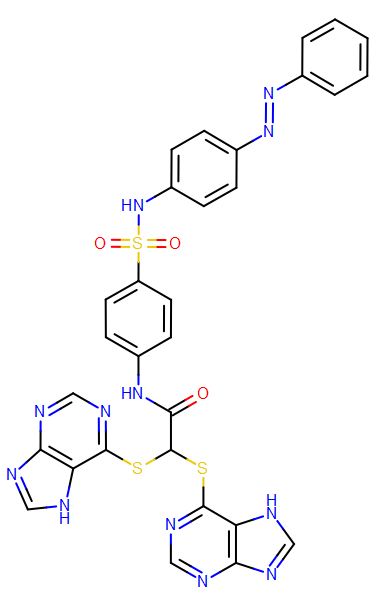  CCR9Ant33, CCR9 |
| 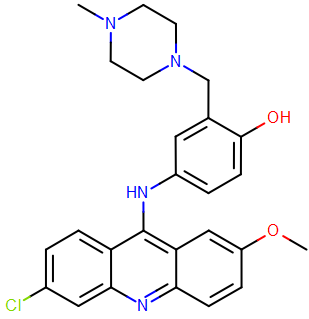  CCR9Ant34, CCR9 | 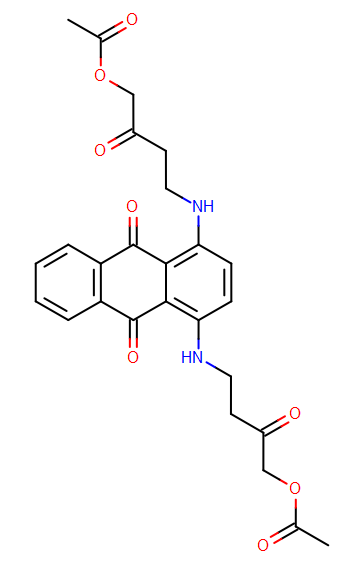  CCR9Ant35, CCR9 | 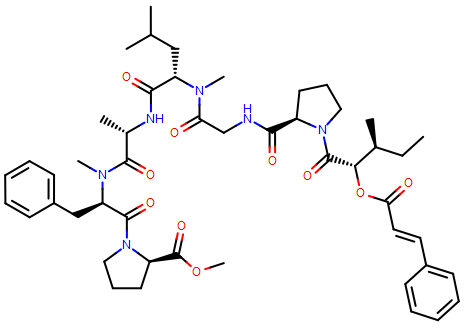  Brintonamide D, CCR10 (ACKR2), CXCR7 | 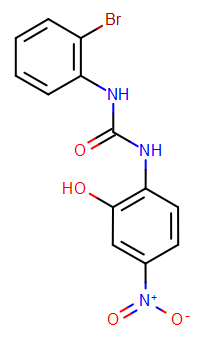  SB225002, CXCR2 |
| 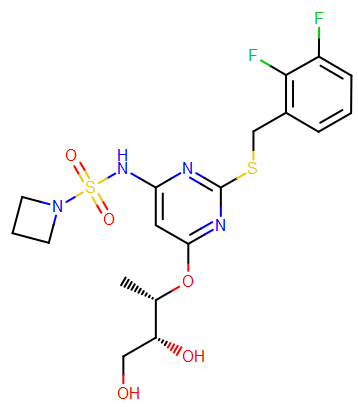  AZD 5096, CXCR2 | 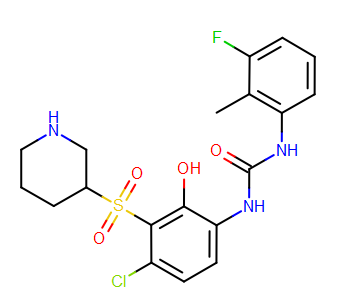  Danirixin, CXCR2 | 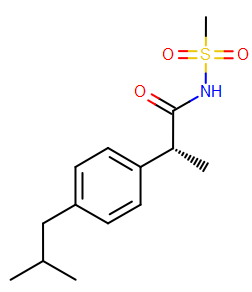  Reparixin, CXCR1, CXCR2 | 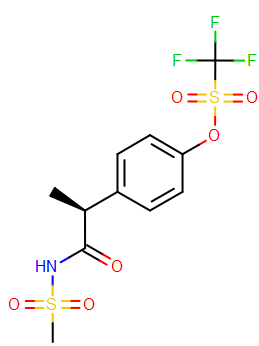  Ladarixin, CXCR2 |
| 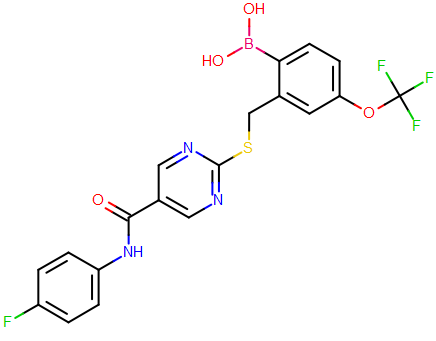  SX682, CXCR2 | 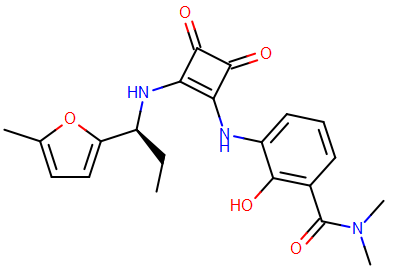  MK7123, CXCR2 | 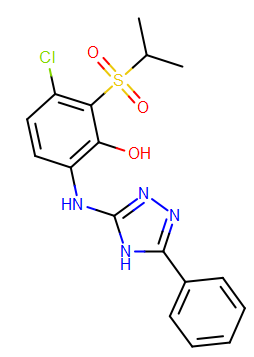  CXCR2Ant5, CXCR2 | 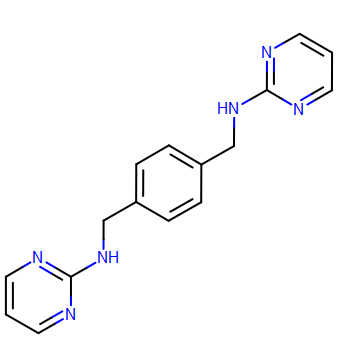  MSX122, CXCR4 |
| 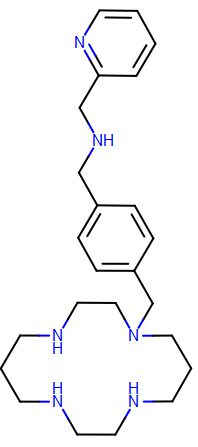  AMD3465, CXCR4 | 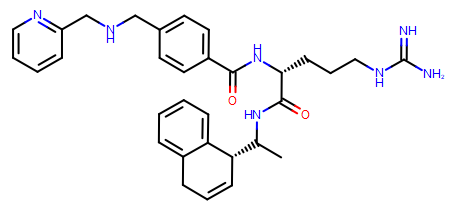  KRH1636, CXCR4 | 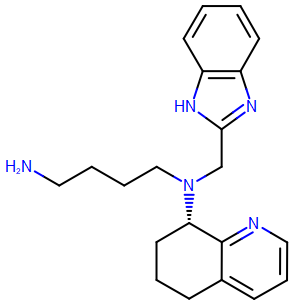  AMD11070, CXCR4 | 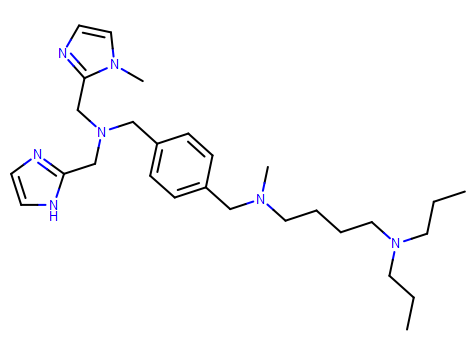  KRH3955, CXCR4 |
| 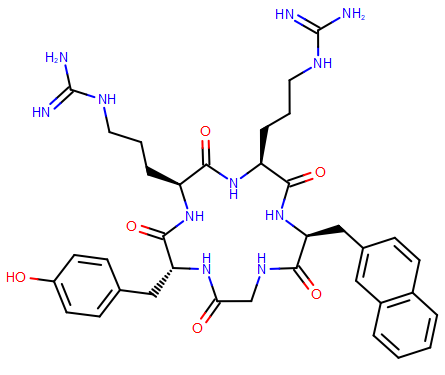  FC131, CXCR4 | 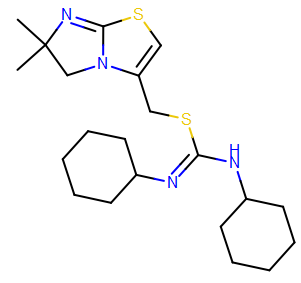  IT1t, CXCR4 | 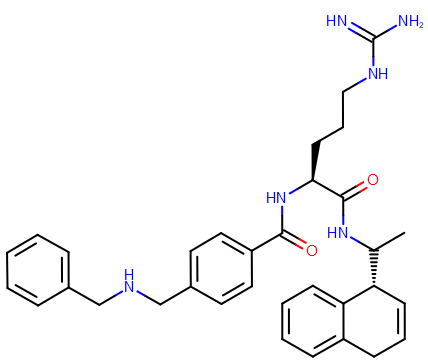  Zach5, CXCR4 | 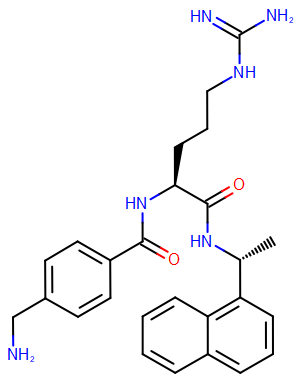  Zach6, CXCR4 |
| 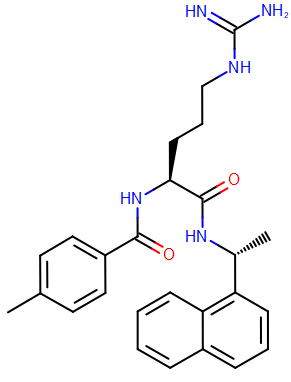  Zach7, CXCR4 | 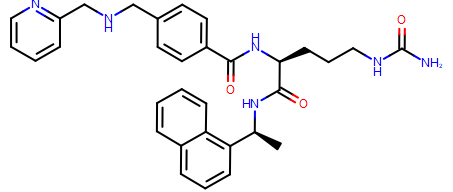  Zach8, CXCR4 | 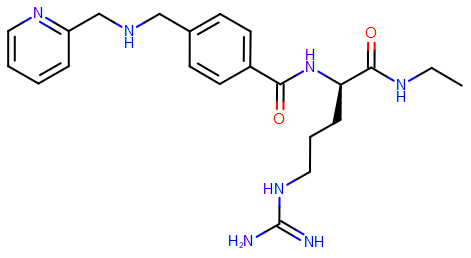  Zach9, CXCR4 | 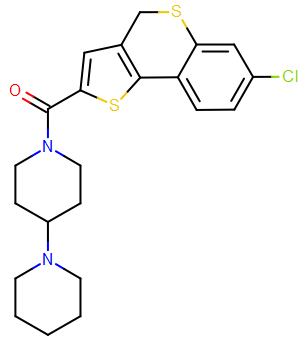  CXCR7Ant1, CXCR7 |
| 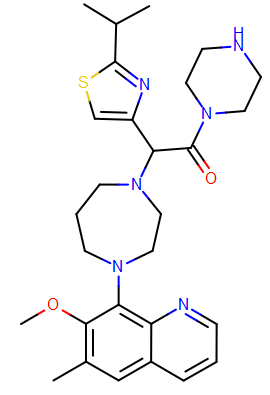  CXCR7Ant2, CXCR7 | 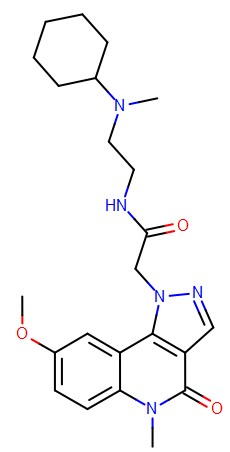  CXCR7Ant3, CXCR7 | 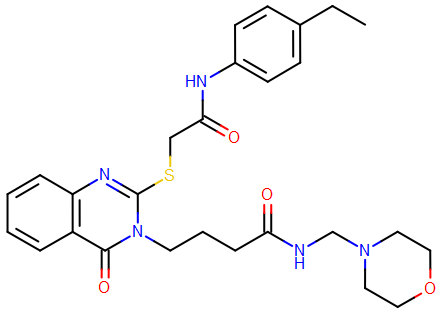  CXCR7Ant4, CXCR7 | 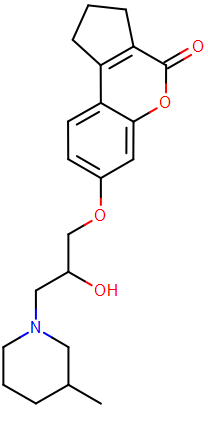  CXCR7Ant5, CXCR7 |
| 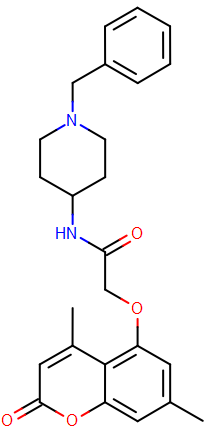  CXCR7Ant6, CXCR7 | 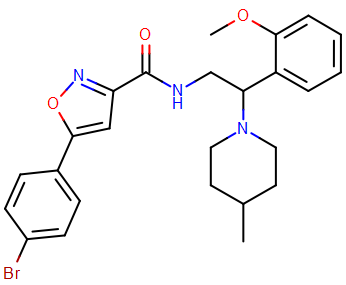  CXCR7Ant7, CXCR7 | 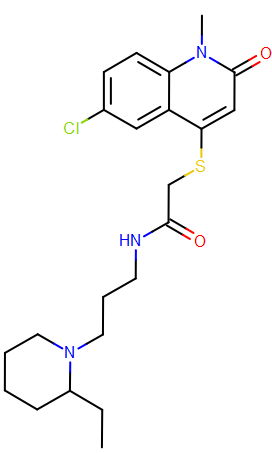  CXCR7Ant8, CXCR7 | 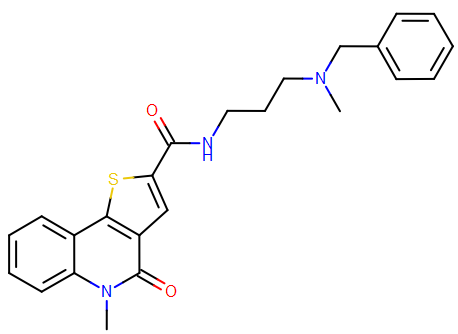  CXCR7Ant9, CXCR7 |
| 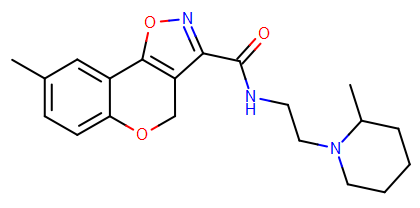  CXCR7Ant10, CXCR7 | 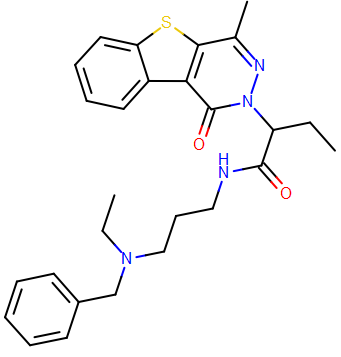  CXCR7Ant11, CXCR7 | 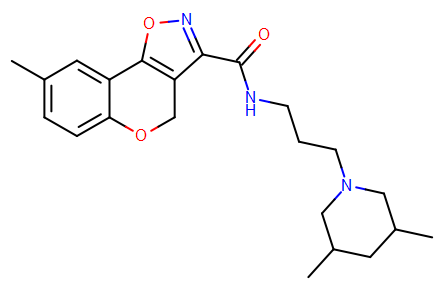  CXCR7Ant12, CXCR7 | 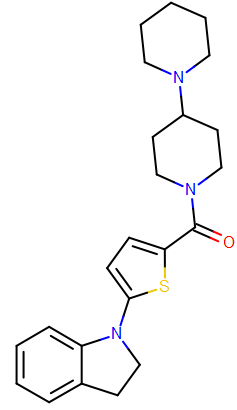  CXCR7Ant13, CXCR7 |
| 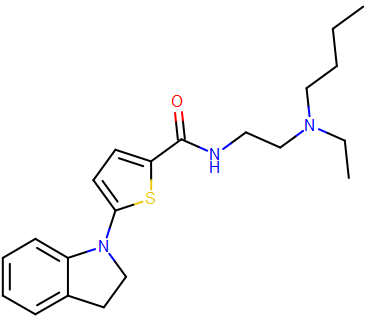  CXCR7Ant14, CXCR7 | 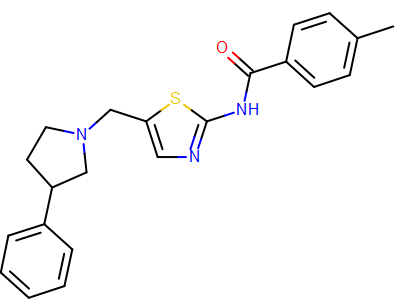  CXCR7Ant15, CXCR7 | 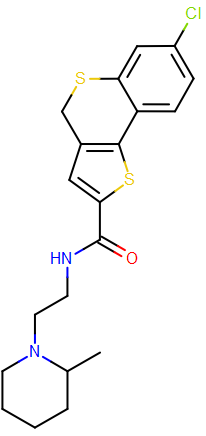  CXCR7Ant16, CXCR7 | 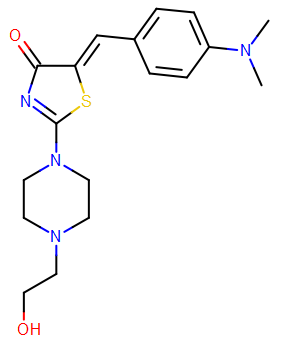  CXCR7Ant17, CXCR7 |
| 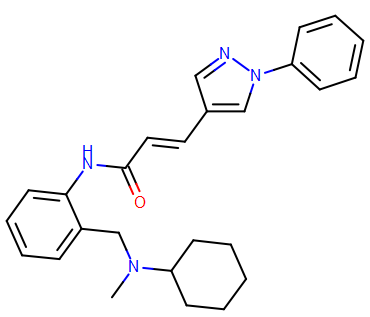  CXCR7Ant18, CXCR7 | 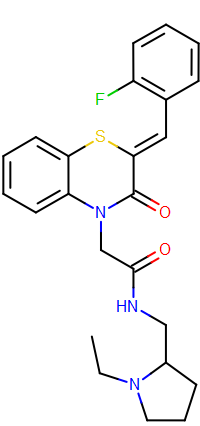  CXCR7Ant19, CXCR7 | 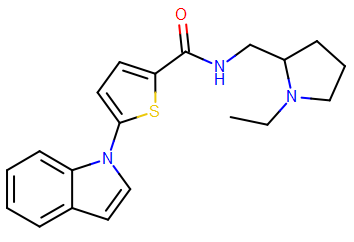  CXCR7Ant20, CXCR7 | 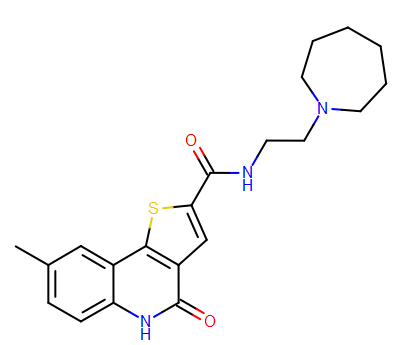  CXCR7Ant21, CXCR7 |
| CXCR7Ant22, CXCR7 | CXCR7Ant23, CXCR7 | CXCR7Ant24, CXCR7 | CXCR7Ant25, CXCR7 |

## Chemistry – General Information

Compounds from libraries of commercially available compounds were obtained from various sources (AA Blocks, ChemBridge, ChemDiv, Enamine, Life Chemicals, Maybridge, Otava, Specs, UkrOrgSynthesis, and Vitas-M) and used as received. The purity of all compounds was above 90%, according to the suppliers. Purity of compounds from the FKKTlib academic compound library was determined to be above 90% (except for compound **C019**), see Supplementary Excel File.

The reagents and solvents were used as received from commercial suppliers. Reactions were monitored using analytical thin-layer chromatography (TLC) on silica gel 60 F_254_ Al plates. Developed plates were inspected under UV light and, if necessary, visualized with ninhydrin, vanillin/sulfuric acid, Dragendorff’s or potassium permanganate stains. Melting points were determined with Büchi 535 Melting Point Appartus (uncorrected). Nuclear magnetic resonance spectra were recorded on a Bruker Avance III 500 MHz and Bruker Avance III 400 MHz spectrometers at 500 MHz (400 MHz) for ^1^H, 126 MHz (101 MHz) for ^13^C, respectively, using DMSO-*d*_6_ or CDCl_3_ with TMS as the internal standard, as solvents. Chemical shifts are reported in *parts per million* (ppm), TMS peak was calibrated to 0 ppm or, alternatively, the central peak of the residual solvent resonance was used as the internal standard, *i.e.* for CDCl_3_ at 7.27 ppm for ^1^H and 77.16 ppm for ^13^C and DMSO-*d*_6_ at 2.50 ppm for ^1^H and 39.52 ppm for ^13^C, respectively. The multiplicities are reported as follows: *s* (singlet), *d* (doublet), *t* (triplet), *q* (quartet), *m* (multiplet), *dd* (doublet of doublets), *ddd* (doublet doublet of doublets), *td* (triplet of doublets), *qd* (quartet of doublets), and *br* (broad), number of equivalent nuclei (by integration), coupling constants (*J*) quoted in Hertz (Hz). Mass spectra were recorded on Agilent 6224 Accurate Mass TOF LC/MS and Thermo Scientific Q Executive Plus LC-MS/MS spectrometers, and IR spectra on Thermo Nicolet FT-IR spectrophotometer. UHPLC analyses were performed on Thermo Scientific Dionex UltiMate 3000 modular system (Thermo Fisher Scientific Inc.) with Waters Acquity UPLC^®^ HSS C18 SB column (2.1 × 50 mm, 1.8 µm) thermostated at 40 °C, injection volume, 1–5 µL; sample, 0.1–0.2 mg/mL in MeOH; flow rate, 0.4 mL/min; detector λ, 220 and 254 nm; mobile phase A: 0.1% TFA (v/v) in water; mobile phase B: MeCN. Method I: 0–5 min, 20%–100% B; 5–6 min, 20% B. Method II: 0–2 min, 0% B; 2–8 min, 0%–100% B. Method III: 0–2 min, 20% B; 2–5 min, 20%–90% B; 5–8 min, 90% B.

### 3-((2-((2-Methoxyethyl)amino)-3,4-dioxocyclobut-1-en-1-yl)amino)benzonitrile (**CS-2**)

3-((2-Ethoxy-3,4-dioxocyclobut-1-en-1-yl)amino)benzonitrile (Mejuch et al., 2017) (230 mg, 0.945 mmol) and 2-methoxyethylamine (1.1 eq., 90 µL) in acetonitrile (5 mL) were stirred for 18h at 60 °C. The reaction mixture was cooled in an icebath for 5 min, the precipitate was filtered, washed with ice-cold acetonitrile (2 x 5 mL), diethyl ether (30 mL) and dried in air to afford 3-((2-((2-methoxyethyl)amino)-3,4-dioxocyclobut-1-en-1-yl)amino)benzonitrile (Jaeger et al., 2019). Yield: 175 mg (0.645 mmol, 67.9%) of yellowish solid. mp = 242.6–243.8 °C. ESI-HRMS: m*/z* = 272.1028 (MH^+^); C_14_H_14_N_3_O_3_ requires: *m/z* = 272.1030 (MH^+^). *ν*_max_ 3269, 3206, 3145, 3090, 3062, 2944, 2234, 1794, 1664, 1607, 1587, 1551, 1489, 1441, 1307, 1112, 1016, 864, 710, 630 cm^–1^. Purity: UPLC (method I, 254 nm): t_r_ = 1.667 min, 96.1% total area. ^1^H NMR (400 MHz, DMSO-*d*_6_): *δ* 3.32 (*s*, 3H), 3.51 (*t*, *J* = 5.0 Hz, 2H), 3.75 – 3.81 (*m*, 2H), 7.45 (*dt*, *J* = 1.1, 7.5 Hz, 1H), 7.53 (*t*, *J* = 7.9 Hz, 1H), 7.61 – 7.66 (*m*, 1H), 7.89 (*s*, 1H), 7.93 (*s*, 1H), 9.94 (*s*, 1H). ^13^C NMR (101 MHz, DMSO-*d*_6_): *δ* 43.49, 58.06, 71.28, 112.15, 118.67, 120.81, 122.55, 125.81, 130.71, 139.99, 162.82, 169.49.

### Ethyl 4-((2-((2-methoxyethyl)amino)-3,4-dioxocyclobut-1-en-1-yl)amino)piperidine-1-carboxylate (**CS-3**)

Ethyl 4-((2-ethoxy-3,4-dioxocyclobut-1-en-1-yl)amino)piperidine-1-carboxylate (Antane et al., 1996) was *in situ* prepared from ethyl 4-aminopiperidine-1-carboxylate (250 mg, 1.45 mmol) and diethyl squarate (1.1 eq., 236 µL) in absolute ethanol (2 mL). After 24h stirring at room temperature, 2-methoxyethylamine (1.5 eq., 190 µL) was added, and the stirring continued for 24h. Ethyl 4-((2-((2-methoxyethyl)amino)-3,4-dioxocyclobut-1-en-1-yl)amino)piperidine-1-carboxylate (Jaeger et al., 2019) was isolated using reversed-phase column chromatography (RP-CC) (Isolera Biotage One Flash Chromatography system, SNAP Biotage KP-C18-HS column, 12 g) using a gradient of 0.1% TFA in deionized water and MeCN as eluent (gradient 10–100% MeCN in 15 column volumes (300 mL); 100% MeCN for 5 column volumes (100 mL)). After the RP-CC, fractions containing the product were combined, and the organic solvent removed *in vacuo*. The remaining aqueous solution was neutralized with saturated sodium bicarbonate solution and extracted with DCM (2 x 30 mL). The combined organic phases were dried over anhydrous sodium sulfate, filtered, and volatile components evaporated in vacuo to afford the pure product. Yield: 365 mg (1.12 mmol, 77.4%) of beige solid. mp = 157.1–158.4 °C. ESI-HRMS: *m/z* = 326.1707 (MH^+^); C_15_H_24_N_3_O_5_ requires: *m/z* = 326.1711 (MH^+^). *ν*_max_ 3221, 2962, 1794, 1669, 1577, 1538, 1431, 1262, 1232, 1147, 1098, 1020, 788, 747, 700 cm^–1^. Purity: UPLC (method I, 254 nm): t_r_ = 3.133 min, 97.1% total area. ^1^H NMR (400 MHz, CDCl_3_): *δ* 1.19 (*t*, *J* = 7.1 Hz, 3H), 1.47 – 1.59 (*m*, 2H), 1.88 – 1.97 (*m*, 2H), 2.89 (*t*, *J* = 10.1 Hz, 2H), 3.27 (*s*, 3H), 3.49 (*t*, *J* = 5.1 Hz, 2H), 3.73 – 3.81 (*m*, 2H), 3.96 – 4.14 (*m*, 5H), 7.62 (*s*, 1H), 7.88 (*s*, 1H). ^13^C NMR (101 MHz, CDCl_3_): *δ* 14.63, 32.84, 42.17, 44.12, 51.41, 58.75, 61.42, 71.84, 155.36, 167.50, 167.85, 181.72, 182.54.

## Supplementary references

Antane, M. M., Butera, J. A., Hirth, B. H., and Antane, S. A. (1996). Diaminocyclobutene-3,4-diones. Available at: https://patents.google.com/patent/US5530025A/en.

Jaeger, K., Bruenle, S., Weinert, T., Guba, W., Muehle, J., Miyazaki, T., et al. (2019). Structural Basis for Allosteric Ligand Recognition in the Human CC Chemokine Receptor 7. *Cell* 178, 1222-1230.e10. doi:10.1016/j.cell.2019.07.028.

Mejuch, T., Garivet, G., Hofer, W., Kaiser, N., Fansa, E. K., Ehrt, C., et al. (2017). Small-Molecule Inhibition of the UNC119–Cargo Interaction. *Angew. Chem. Int. Ed.* 56, 6181–6186. doi:10.1002/anie.201701905.

Taveras, A. G., Chao, J., Biju, P. J., Yu, Y., Aki, C. J., Merritt, J. R., et al. (2010). Thiadiazoledioxides and thiadiazoleoxides as CXC- and CC-chemokine receptor ligands. Available at: https://patents.google.com/patent/US7691856/en?oq=US20070264230A1.
